# Serial-section Electron Tomography and Quantitative Analysis of Microtubule Organization in 3D-reconstructed Mitotic Spindles

**DOI:** 10.21769/BioProtoc.4849

**Published:** 2023-10-20

**Authors:** Robert Kiewisz, Daniel Baum, Thomas Müller-Reichert, Gunar Fabig

**Affiliations:** 1 Simons Machine Learning Center, New York Structural Biology Center, New York, NY, USA; 2 Experimental Center, Medizinische Fakultät Carl Gustav Carus, Technische Universität Dresden, Dresden, Germany; 3 Biocomputing Unit, Centro Nacional de Biotechnologia (CNB-CSIC), Darwin, 3, Campus Universidad Autonoma, Cantoblanco, Madrid, Spain; 4 Department of Visual and Data-Centric Computing, Zuse Institute Berlin, Berlin, Germany

**Keywords:** Microtubules, Mitotic spindle, HeLa cells, Tissue culture cells, Human cells, Serial sectioning, Electron tomography, 3D reconstruction, Automatic segmentation of microtubules, Automated spatial graph analysis, Electron microscopy, Correlative light and electron microscopy, CLEM

## Abstract

For the analysis of cellular architecture during mitosis, nanometer resolution is needed to visualize the organization of microtubules in spindles. Here, we present a detailed protocol that can be used to produce 3D reconstructions of whole mitotic spindles in cells grown in culture. For this, we attach mammalian cells enriched in mitotic stages to sapphire discs. Our protocol further involves cryo-immobilization by high-pressure freezing, freeze-substitution, and resin embedding. We then use fluorescence light microscopy to stage select mitotic cells in the resin-embedded samples. This is followed by large-scale electron tomography to reconstruct the selected and staged mitotic spindles in 3D. The generated and stitched electron tomograms are then used to semi-automatically segment the microtubules for subsequent quantitative analysis of spindle organization. Thus, by providing a detailed correlative light and electron microscopy (CLEM) approach, we give cell biologists a toolset to streamline the 3D visualization and analysis of spindle microtubules (http://kiewisz.shinyapps.io/asga). In addition, we refer to a recently launched platform that allows for an interactive display of the 3D-reconstructed mitotic spindles (https://cfci.shinyapps.io/ASGA_3DViewer/).

Key features

• High-throughput screening of mitotic cells by correlative light and electron microscopy (CLEM).

• Serial-section electron tomography of selected cells.

• Visualization of mitotic spindles in 3D and quantitative analysis of microtubule organization.

**Graphical overview**


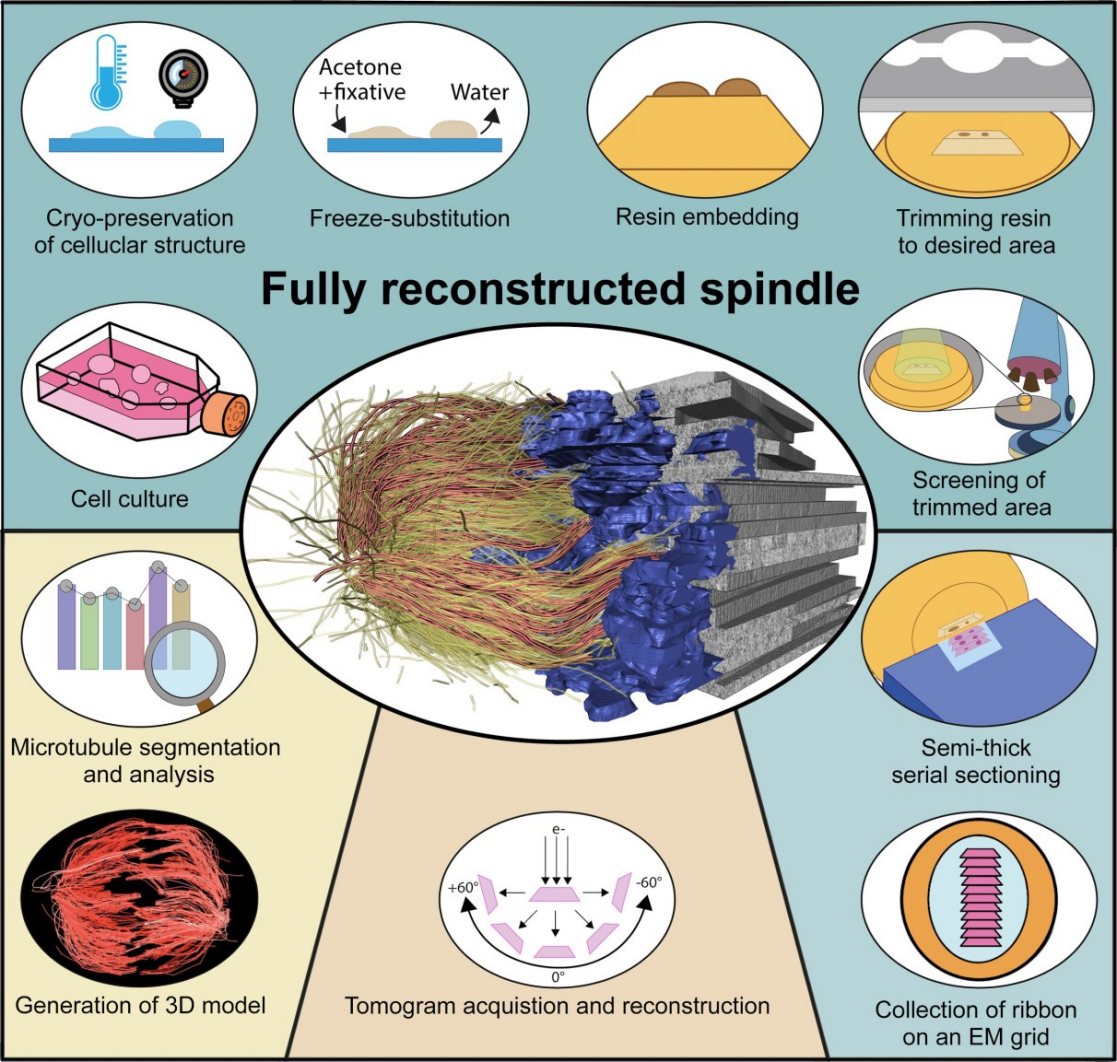


## Background

It is becoming more and more important to collect data on the three-dimensional (3D) architecture of cells in different model systems. For example, in the field of mitosis, it is important to understand the organization of the microtubule cytoskeleton in each mitotic stage. Working with 3D data, however, can be challenging due to a number of specific methodological and computational requirements. Here, we apply serial-section electron tomography for quantitative analysis of spindle organization in mammalian cells in culture. Obtaining 3D data by electron tomography is time consuming and this leads to the generation of large data sets that can be difficult to handle. Electron tomography also requires extensive computational resources. Moreover, analysis of the generated 3D spatial graphs involves complex computational and visualization tools due to the high complexity of the spindle ultrastructure (Müller-Reichert et al., 2003; Kiewisz et al., 2022). There is a clear need for dedicated software tools for analyzing 3D spatial graphs. Such software can help researchers overcome the challenges associated with working with 3D data and accelerate research in the field of mitosis. Recently, progress has been achieved by increasing access to resources for handling and analyzing large data sets.

Our protocol gives step-by-step instructions for sample preparation and data acquisition by electron tomography (O’Toole et al., 2020). In addition, the computational tools for the quantification and visualization of the 3D data will also be described in detail. The workflow presented here has been applied for serial-section large-scale electron tomography of entire mitotic spindles in mitotic HeLa cells (O’Toole et al., 2020; Kiewisz et al., 2022). Our workflow, however, can be applied for the visualization and analysis of any other cell type grown on appropriate support. In addition, we introduce a newly developed platform that allows for quantitative analysis of the 3D spatial graphs, including all segmented microtubules of the 3D-reconstructed spindles (https://cfci.shinyapps.io/ASGA_3DViewer/). This platform, termed *Automated Spatial Graph Analysis* (ASGA) is designed for a flexible online display of spindles with a modular structure that allows for the swift addition of new analysis tools. Using this *3DViewer* within ASGA, segmented spindles can be visualized, quantitatively analyzed, and interactively shared.

## Materials and reagents

**Biological material**

1. HeLa Kyoto cells (Gerlich Lab, IMBA, RRID: CVCL_1922)

**Reagents**

1. Acetone (CH_3_COCH_3_), glass-distilled, EM-grade (EMS, Electron Microscopy Sciences, catalog number: 10015)
2. BSA (albumin fraction V) (Carl Roth, catalog number: 8076.2)
3. Chloroform (CHCl_3_) (EMS, catalog number: 12550)
4. Colloidal gold solution (Ø 20 nm) (BBI, British Biocell International Solutions, catalog number: EM.GC20/4)
5. Disodium hydrogen phosphate (Na_2_HPO_4_) (Carl Roth, catalog number: P030.1)
6. DMEM (Dulbecco’s Modified Eagle Medium, 1×) + GlutaMax^TM^-I (Thermo Fisher Scientific, catalog number: 10566016)
7. DMSO (Thermo Fisher Scientific, catalog number: D4540-100ML)
8. Ethanol absolute (C_2_H_5_OH) (VMR, catalog number: 20821.296)
9. FBS (fetal bovine serum) (HI), origin: USA (Thermo Fisher Scientific, catalog number: 10082147)
10. Fibronectin (Sigma-Aldrich, catalog number: F0556-100UL)
11. Formvar (Science Services, catalog number: E15800)
12. High-vacuum grease (Thorlabs Elliptec GmbH, catalog number: SG10)
13. Hydrogen peroxide 30% (Merck KGaA, catalog number: 1072100250)
14. Lead citrate (C_12_H_10_O_14_Pb_3_) (Science Services, catalog number: E17810)
15. Methanol (CH_3_OH) (VWR, catalog number: 20834.291)
16. Methylene blue (C_16_H_18_ClN_3_S) (Carl Roth, catalog number: A514.1)
17. Osmium tetroxide (OsO_4_) (Science Services, catalog number: E19100)
18. Penicillin/Streptomycin (Thermo Fisher Scientific, catalog number: 10378016)
19. Poly-L-lysine (0.01%) (Sigma-Aldrich, catalog number: A-005-C)
20. Potassium chloride (KCl) (AppliChem GmbH, catalog number: 31494.1210)
21. Potassium dihydrogen phosphate (KH_2_PO_4_) (Carl Roth, catalog number: 3904.1)
22. Propanol (C_3_H_7_OH) (VWR, catalog number: 20858.293)
23. Sodium chloride (NaCl) (Carl Roth, catalog number: HN00.2)
24. Sulfuric acid 96% (H_2_SO_4_) (AppliChem GmbH, catalog number: 131058.1211)
25. Trypsin-EDTA 1× (0.25%) (Thermo Fisher Scientific, catalog number: 25200056)
26. Uranyl acetate [UO_2_(CH_3_COO_2_)] (Polysciences, catalog number: 21447-25)

**Solutions**

1. AFS (automatic freeze substitution) *cocktail* containing methylene blue (see Recipes)
2. DMEM-WT (standard medium for mammalian cell culture) (see Recipes)
3. DMEM-HPF (high-pressure freezing) solution (see Recipes)
4. Epon/Araldite resin (see Recipes)
5. Fibronectin coating solution (see Recipes)
6. Formvar solution (see Recipes)
7. Lead citrate post-staining solution (see Recipes)
8. PBS 10× buffer (see Recipes)
9. Uranyl acetate post-staining solution (see Recipes)

**Recipes**

1. **AFS (automatic freeze substitution) *cocktail* containing methylene blue**
  Oversaturate 60 mL of acetone from a freshly opened bottle of glass-distilled, anhydrous acetone with methylene blue by adding 5 g to it and mixing it by shaking it several times at room temperature. After placing the closed bottle at -80 °C overnight, filter the solution with a syringe filter. Then, use the filtered acetone for the preparation of the AFS *cocktail* as described below. Aliquot 1 mL each in 2 mL cryo-tubes, freeze them immediately in liquid nitrogen, and store them in liquid nitrogen until further use.
  **Table:** 

Reagent	Final concentration	Quantity
Osmium tetroxide	1% (w/v)	0.5 g
Uranyl acetate	0.1% (w/v)	0.05 g
Acetone (saturated with methylene blue)	n/a	50 mL
Total	n/a	50 mL
2. **DMEM-WT (standard medium for mammalian cell culture)**
  **Table:** 

Reagent	Final concentration	Quantity
FBS	9.01% (v/v)	50 mL
Penicillin/Streptomycin	0.9% (v/v)	5 mL
DMEM	n/a	500 mL
Total	n/a	555 mL
3. **DMEM-HPF (high-pressure freezing) solution**
  **Table:** 

Reagent	Final concentration	Quantity
BSA	10% (w/v)	1 g
DMEM-WT	n/a	10 mL
Total	n/a	10 mL
4. **Epon/Araldite resin**
  Mix all reagents in a single-use plastic beaker with a magnetic stirrer overnight. The next day, fill up 10 mL syringes with mixed resin, close them with Parafilm, and store them at -20 °C until further use.
  **Table:** 

Reagent	Final concentration	Quantity
Embed 812	n/a	25 mL
Araldite 502	n/a	15 mL
DDSA	n/a	55 mL
BDMA	n/a	2.5 mL
Total	n/a	97.5 mL
5. **Fibronectin coating solution**
  **Table:** 

Reagent	Final concentration	Quantity
Fibronectin	n/a	0.1 mL
PBS (1×)	n/a	0.9 mL
Total	n/a	1 mL
6. **Formvar solution**
  **Table:** 

Reagent	Final concentration	Quantity
Formvar powder	1% (w/v)	1 g
Chloroform	n/a	100 mL
Total	n/a	100 mL
7. **Lead citrate post-staining solution**
  **Table:** 

Reagent	Final concentration	Quantity
Lead citrate	0.4% (w/v)	2 g
Double-distilled water	n/a	50 mL
Total	n/a	50 mL
8. **PBS (10×) buffer**
  **Table:** 

Reagent	Final concentration	Quantity
Na_2_HPO_4_	100 mM	8.9 g
KH_2_PO_4_	18 mM	1.2 g
NaCl	1.37 M	40 g
KCl	27 mM	1 g
Double-distilled water	n/a	500 mL
Total	n/a	500 mL
9. **Uranyl acetate post-staining solution**
  **Table:** 

Reagent	Final concentration	Quantity
Uranyl acetate	2% (w/v)	0.2 g
Methanol	n/a	10 mL
Total	n/a	10 mL

**Laboratory supplies**

1. Beaker, single-use plastic (VWR, catalog number: 414004-148)
2. Compressed air bottle (Falcon Safety Products UK Limited, catalog number: 88004/DPSRX/UK)
3. Coverslips (24 mm × 60 mm) (Marienfeld, catalog number: 0107242)
4. Cryo-tube (1.5 mL) (Greiner Bio-One, catalog number: 126261)
5. Desmotome VT1, straight (Ustomed, catalog number: 83-898-000)
6. Epon/Araldite (Araldite 502/Embed 812/DDSA/BDMA kit) (Science Services GmbH, catalog number: 13940)
7. Eppendorf tubes (0.5, 1, and 2 mL) (Eppendorf, catalog number: 0030121589, 0030120094)
8. Falcon tubes (15 and 50 mL) (Sigma-Aldrich, catalog number: T1943, T2318)
9. Filter paper (Sigma-Aldrich, catalog number: WHA1540090)
10. Flow-through rings (Leica, catalog number: 16707157)
11. Glass slides (76 mm × 26 mm) (Engelbrecht, catalog number: 11101)
12. Grid staining system (PELCO^®^ Staining slot matrix) (Ted Pella, catalog number: 22510)
13. Grid staining system (PELCO^®^ Staining vessels) (Ted Pella, catalog number: 22510-2)
14. Grids (oval slot 2 mm) (EMS, catalog number: G2010-Cu)
15. Nunc^TM^ EasYFlask^TM^ (T-75 and T-175) (Thermo Fisher Scientific, catalog number: 156499, 159910)
16. Parafilm (Pechiney, catalog number: HS234526A)
17. PHA/PLA 3D printer filament (1.75 mm), natural color (ColorFabb, catalog number: 010003)
18. Planchettes for high-pressure freezing (Ø 3 mm, indentation 40/600 μm) (Wohlwend GmbH, catalog number: Art. 737)
19. Precision wipes (KimTech) (Kimberly-Clark, catalog number: 75512)
20. Razor blades (double edge, coated) (EMS, catalog number: 72000)
21. Razor blades (single edge) (Plano GmbH, catalog number: T586)
22. Reagent bath (Leica, catalog number: 16707154)
23. Sapphire discs for high-pressure freezing (Ø 3 mm, thickness 0.16 mm) (Wohlwend GmbH, catalog number: 500)
24. Syringe (1 mL) (Braun, catalog number: 9166017V-02)
25. Syringe (10 mL) (Braun, catalog number: 4617100V-02)
26. Syringe filter (0.2 μm mesh size) (Sarstedt AG, catalog number: 83.1826.001)
27. Syringe needle (25 G, nr. 18) (Becton Dickinson S.A., catalog number: 305125)
28. Syringe needle (27 G, nr. 20) (Becton Dickinson S.A., catalog number: 305109)
29. Tweezers (style 5X) (EMS, catalog number: 78320-5X)
30. Tweezers (style SS) (EMS, catalog number: 78325-SS6)

## Equipment

1. 3D printer (Prusa i3 MK2s) (Prusa Research, catalog number: i3-MK2s)
2. 3D printer nozzle (V6, Stainless-steel nozzle, 1.75 mm) (E3D)
3. CCD camera (F214), mounted on a TECNAI T12 TEM (TVIPS GmbH)
4. CCD camera (US1000), mounted on a TECNAI F30 TEM (Gatan)
5. Cell culture laminar flow cabinet Heraeus (HERASAFE HSP12) (Heraeus)
6. Centrifuge (5702 R) (Eppendorf, catalog number: 5703000010)
7. Diamond knife (Ultra 35°) (Diatome, catalog number: DU3515)
8. Dual-axis TEM holder (Fischione, model: 2040)
9. Formvar film casting device (EMS, catalog number: 71305-01)
10. Freezer (MDF U55V, -80 °C) (Sanyo)
11. High-pressure freezer (Compact 03) (Wohlwend GmbH)
12. Incubator HERP (Cell 240) (Thermo Fisher Scientific, catalog number: 51026331)
13. Incubator HPF (TECO 20) (Selutec, catalog number: 600.005)
14. Leica EM AFS2 (Leica)
15. Leica SP5 confocal microscope (Leica)
16. Multitool, Fortiflex (Dremel, catalog number: 9100-21)
17. Objective Leica (HCX PL APO lambda blue 63× 1.2 water) (Zeiss)
18. Objective Leica (HCX PL APU CS 20× 0.7 dry) (Zeiss)
19. Objective Zeiss (A-Plane (44 10 20), 5×/0.12) (Zeiss)
20. Objective Zeiss (A-Plane (44 10 51), 40×/0.65) (Zeiss)
21. Oven (UNB200, 60 °C) (Memmert, catalog number: GZ-52200-01)
22. Scale (BP 110s) (Sartorius)
23. Stereomicroscope (SMZ-168) (Motic, catalog number: 1100200500454)
24. Transmission electron microscope (TEM) (TECNAI F12) (Thermo Fisher Scientific)
25. Transmission electron microscope (TECNAI F30) (Thermo Fisher Scientific)
26. Ultramicrotome Leica (EM UC6) (Leica)
27. Warm bath (GFL 14L) (GFL, catalog number: 3340)

## Software and datasets

1. ASGA (v0.37.0, 25.04.2022)
2. ASGA-3DView (v1.3.1, 03.08.2022)
3. Automatization scripts for tomogram reconstructions (n/a, 08.11.2020)
4. EMMenu (4.0.8.16, n/a)
5. ImageJ/Fiji (v1.52v, 14.04.2020)
6. IMOD (v4.8.22 - 4.12.40, n/a - 18.03.2023)
7. Amira (2022.2, 20.11.2022)
8. AmiraZIBEdition (2023.12, 19.04.2023)
9. R (v4.00 - 4.2.3, 04.2020 - 03.2023)
10. SerialEM (v3.0.2 - 3.8.0beta, n/a)

## Procedure

- **Start of the cell culture**
  Mammalian cells are cultured in DMEM-WT. Cryopreserved cells are used to initiate the culture.
  1. Quickly thaw the cryotube with cells in a warm water bath.
  2. Add thawed cells to a T75 flask with 10 mL of pre-warmed DMEM-WT medium.
  3. Incubate cells for one day at 37 °C with 5% CO_2_ in a humidified incubator.
  4. The next day, replace the medium with a fresh pre-warmed DMEM-WT medium.
  5. Continue cell passage 2–3 times to ensure the culture is healthy.
  6. The day before the initial experiment, ensure that the cell colony achieves approximately 70% confluency.
- **High-pressure freezing**
  1. 3D-printed chambers for high-pressure freezing:
    Mitotic cells are highly sensitive and must be handled with care to avoid any interference with the mitotic process. To avoid any difficulties associated with the cryo-preservation of mitotic cells, we developed custom-made chambers using 3D printing technology to plate cells on a solid support. We designed 3D-printed chambers for small sapphire discs (3 mm in diameter). These chambers are designed to provide a stable environment for mitotic cells for a short time period before high-pressure freezing. Due to their small volume, these chambers increase the number of cells attached to the sapphire discs, thus enabling one to collect cells of the desired mitotic stages (Figure 1; Files S1 and S2 for 3D models). The chamber can hold either one (single chamber) or up to four sapphire discs (quadruple chamber). Before use, the 3D-printed chambers are cleaned with ethanol and stored in a humidified incubator at 37 °C with 5% CO_2_. For a high-throughput approach, we suggest using the quadruple chambers as they allow for an increased yield of collected mitotic cells.
    **Figure 1.**
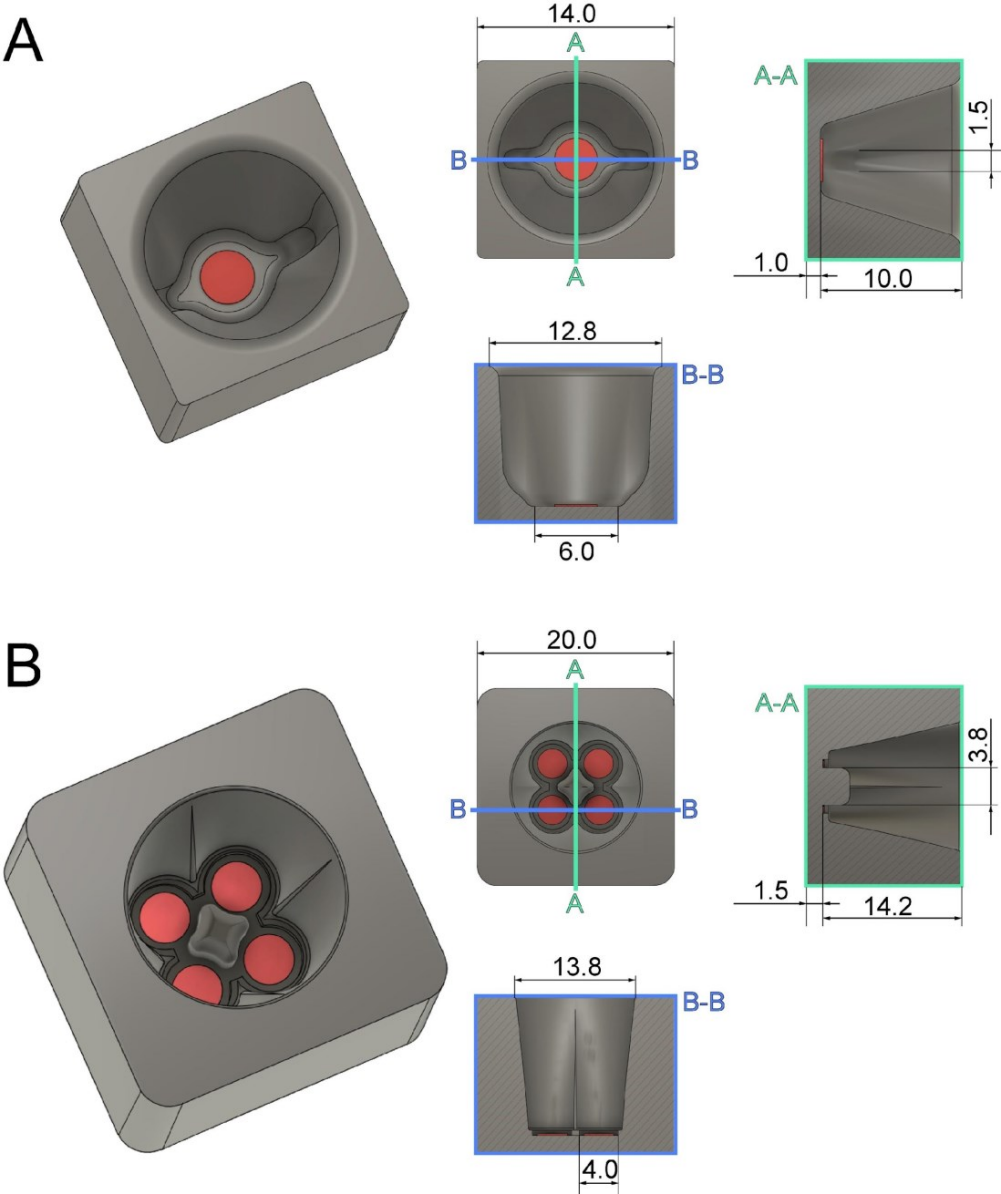
Technical drawing of custom-designed and 3D-printed incubation chambers optimized for attachment of mitotic cells to sapphire discs (Ø 3 mm, red) after shake-off. This chamber design allows for maximizing the yield of attached cells on a small surface area of the sapphire disc. All dimensions are given in millimeters. (A) Culture chamber for a single sapphire disc optimized for small-volume experiments. (B) Culture chamber for four sapphire discs optimized for high-throughput experiments. This figure was reproduced with permission from the book chapter “High-throughput screening of mitotic mammalian cells for electron microscopy using classic histological dyes”, Methods in Cell Biology (162), 151–170 (2021), Copyright Elsevier [Kiewisz et al. (2021)].
  2. Preparation of sapphire disks: Sapphire discs were chosen for cryo-immobilization of cells due to their high thermal conductivity.
    - Cleanup: clean sapphire disc in pure acetone followed by washing in pure ethanol and extensive washing and storage in ddH_2_O for further use (Figure 2A).
      **Figure 2.**
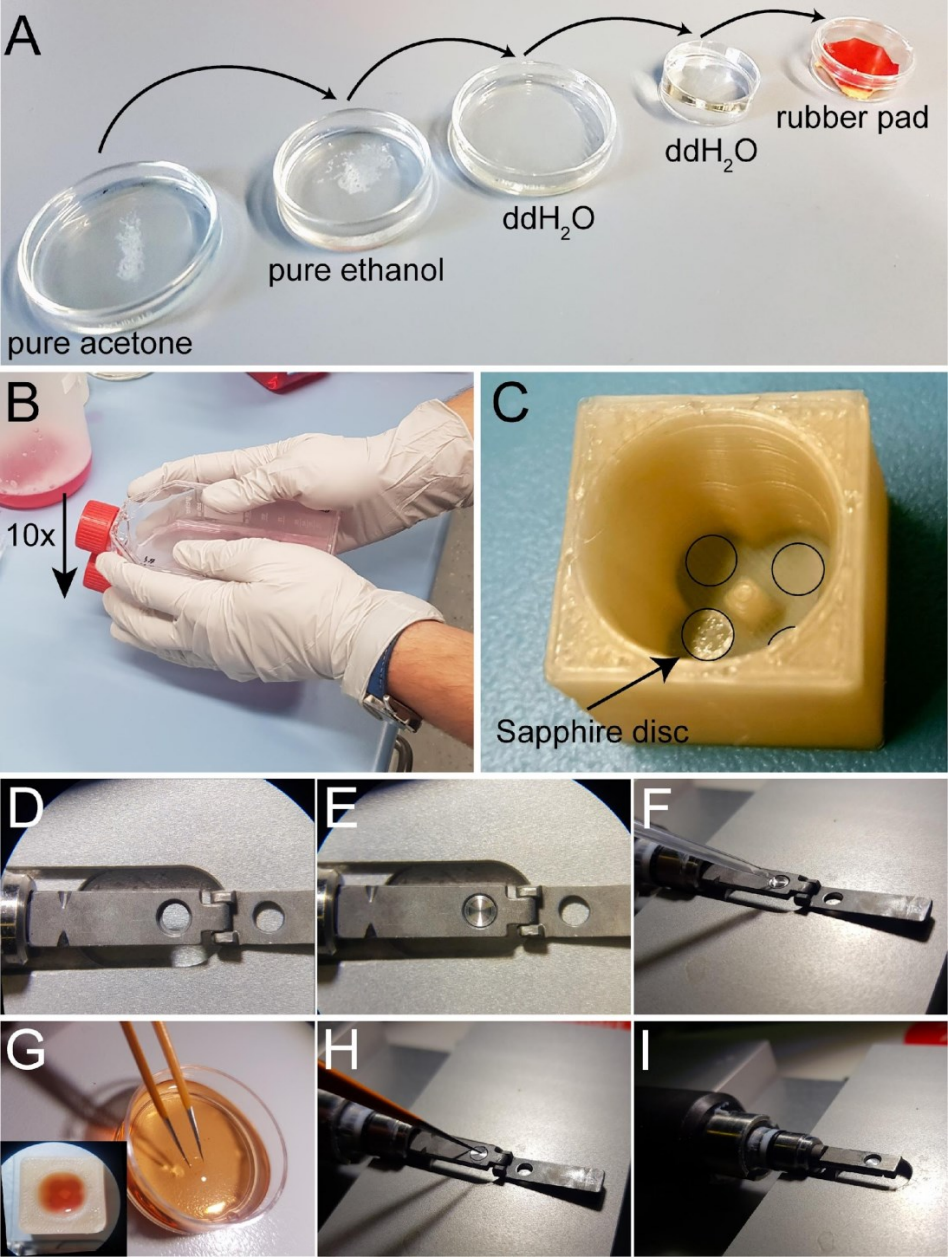
Attachment of mitotic cells on sapphire discs and assembly of *sandwiches* for high-pressure freezing. (A) Cleaning steps for the sapphire discs. (B) Collection of mitotic cells by applying the *shake-off* technique. One or multiple flasks are hit ten times to the surface of the bench. (C) Placement of coated sapphire discs into the 3D-printed incubation chamber designed for four samples. Transparent coated sapphire discs are indicated with black circles. (D) Top view of the opened holder for high-pressure freezing. (E) Placing the aluminum carrier into the sample holder in preparation for freezing. (F) Prefilling the carrier with the freezing medium. (G) Removing a sapphire disc from the quad incubation chamber (lower left corner) with the help of tweezers. This is followed by a quick dipping of the sample into the freezing medium. (H) Placing the sapphire disc onto the aluminum carrier (cells facing down). (I) Closing of the holder to immediately start high-pressure freezing. This figure was reproduced with permission from the book chapter “High-throughput screening of mitotic mammalian cells for electron microscopy using classic histological dyes”, Methods in Cell Biology (162), 151–170 (2021), Copyright Elsevier [Kiewisz et al. (2021)]. See also Video 1.
      **Video 1.**
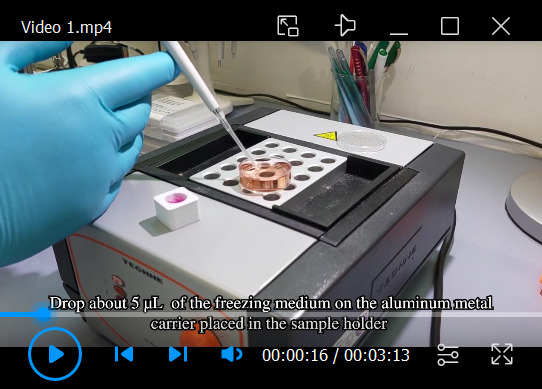
Video tutorial showing the assembly of a *sandwich* for cryo-immobilization using the Wohlwend Compact 03 high-pressure freezer. This video corresponds to Figures 2 and 3.
      **Figure 3.**
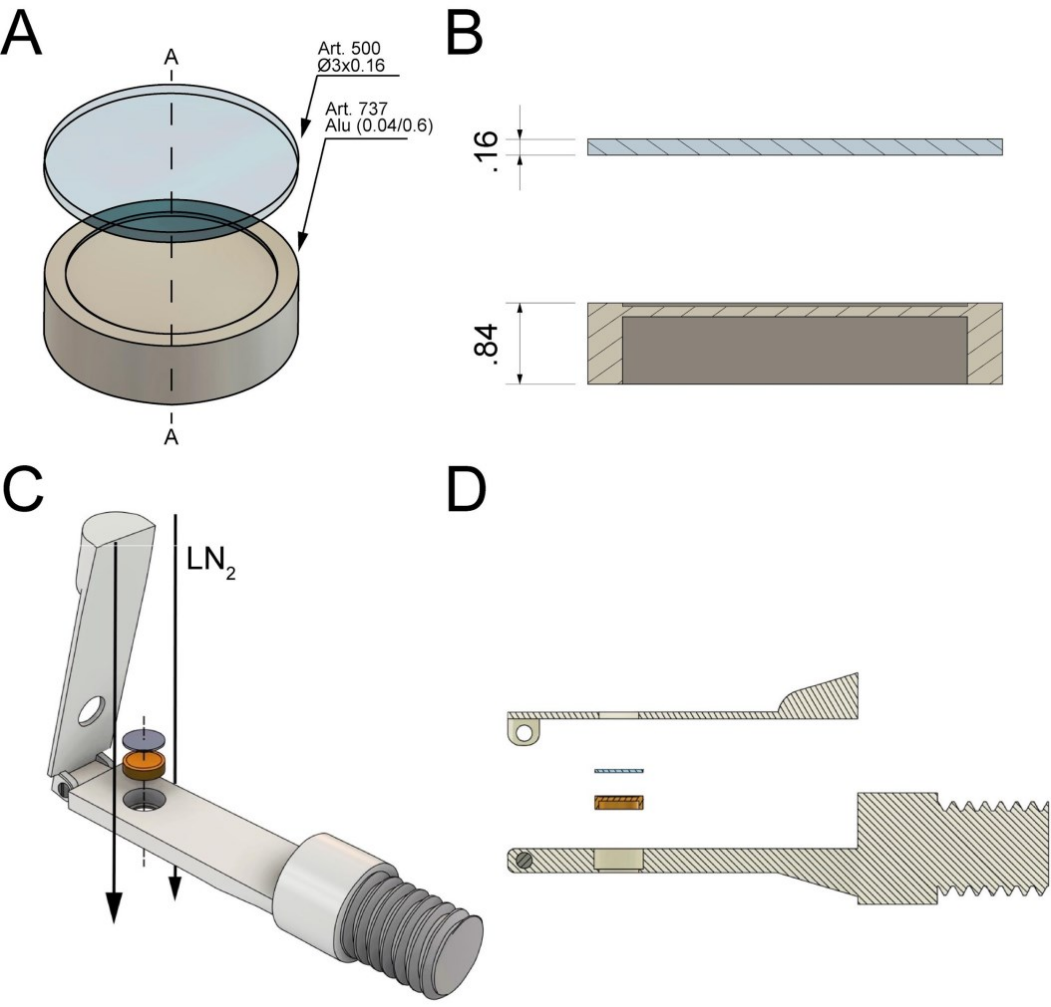
Technical drawing of the *sandwich* as used to cryo-immobilize mitotic cells attached to the sapphire by using the Wohlwend Compact 03 high-pressure freezer. All dimensions are given in millimeter. (A) A sapphire disc (with the attached cells facing down, not shown here) is placed on top of an aluminum carrier with a cavity of 0.04 mm. (B) Cross-section views (A-A as shown in A) of the sample holders. (C) Schematic representation of *sandwich* assembly and insertion of the sample into the sample holder. The flow of the LN_2 _during high-pressure freezing is indicated by black arrows. (D) Side view of the sample loading and the *sandwich* components (sapphire disc and aluminum planchette). This figure was reproduced and modified with permission from the book chapter “High-throughput screening of mitotic mammalian cells for electron microscopy using classic histological dyes”, Methods in Cell Biology (162), 151–170 (2021), Copyright Elsevier [Kiewisz et al. (2021)].
    - Place all sapphire discs into a glass beaker with the cleaning solution and gently mix it to ensure that all sapphire discs are separated. Let it incubate for approximately 5–10 min.
    - After the incubation time, remove all sapphire discs with fine tweezers and rinse them in water two times by placing them in three dishes with ddH_2_O and then once in ethanol (70%, v/v). Store them in an Eppendorf tube in 70% ethanol until further use.
    - Coating: after the cleanup, coat the sapphire discs with poly-L-lysine (0.1% in ddH_2_O, w/v) and fibronectin (1:10 dilution in 1× PBS, v/v).
    - Initially, slowly air dry the sapphire discs by placing them for 1 h in a closed Petri dish to prevent any contamination. Then, coat the discs with poly-L-lysine by applying a single drop on each disc and leaving it for 5 min under a Petri dish.
    - After that, absorb the excess solution with small pieces of filter paper and then place the sapphire discs on a heating plate set to 60 °C for 2 h.
    - Remove sapphire discs from the heating plate and leave them to cool down in a closed Petri dish. It helps to place the discs on a slightly sticky rubber pad (e.g., a piece of table tennis rubber or rubber glove). This will prevent the discs from sticking to a pipette tip.
    - Next, coat the sapphire discs with fibronectin. Prepare a fresh 10% (v/v) fibronectin solution by diluting fibronectin in 1× PBS and gently mixing the solution with a pipette (the solution should not be mixed extensively to prevent fibronectin aggregation).
    - Finally, apply a drop of fibronectin solution on the sapphire discs and leave them at room temperature for 10 min. After that, remove the droplet with a pipette.
    - Place the sapphire discs into custom-designed 3D-printed chambers and store them in the cell culture incubator until further use.
  3. Mitotic shake-off: our preferred method for enriching mitotic cells is called a *mitotic shake-off* (Guizetti et al., 2011; Ma and Poon, 2017). This method takes advantage of the fact that mitotic cells round up during mitosis and can be easily detached. This approach is preferred because it does not require the use of any drugs and reduces temperature stress that may influence mitosis processes (Rao and Engelberg, 1965; Figure 2B).
    - Warm the culture medium to 37°C and store it open in the incubator to allow for saturation with 5% CO_2_.
    - If possible, move all T75 flasks with cell cultures to the incubator located conveniently in the same room as your high-pressure freezer machine one day before the planned experiment. This will limit the number of environmental changes during the mitotic shake-off.
    - To synchronize the cells, remove the cell culture flask from the incubator and hit the bottom of the flask vigorously on a solid surface (e.g., the bench).
    - Remove the entire medium from the flask that contains primarily dead and already mitotic cells and wash it by adding 5 mL of pre-warmed medium.
    - Discard the medium from the flask, add 10 mL of fresh pre-warmed medium, and immediately place it back in the incubator for the next 2 h.
    - Repeat steps B3c–B3e for multiple flasks at approximately 15–20 min intervals if multiple rounds of cryo-immobilization are needed.
    - After 2 h of incubation time, repeat step B3c for the first flask.
    - This time, collect all the medium after the shake-off with mainly mitotic cells by aspirating it with a 15 mL pipette, and place it in a single 15 mL Falcon tube.
    - Centrifuge collected medium gently at 0.2× *g* for 5 min at 37 °C. After this step, one should be able to see a small cell pellet at the bottom of the Falcon tube.
    - Remove the entire supernatant. Be very careful to not remove the cell pellet.
    - Resuspend the cells with 1 mL of pre-warmed medium, mix it, and immediately add it to the sapphire discs stored in the 3D-printed chamber (Figure 2C).
    - Immediately move the 3D-printed chamber to a humidified incubator at 37 °C with 5% CO_2_ for 10–15 min (depending on the mitotic stage that should be analyzed and on the cell type) to allow for the reattachment of the cells to the sapphire discs.
  4. Sandwich assembly for high-pressure freezing and cryo-immobilization:
    For optimal cryo-immobilization and ultrastructural preservation of the samples, high-pressure freezing was applied (McDonald and Morphew, 1993). We used the Wohlwend Compact 03 high-pressure freezer (Figure 3; Video 1). However, any other high-pressure freezer could be used for cryo-immobilization of mammalian cells.
    - Warm up a metal heating block to 37 °C.
    - Remove the 3D-printed chamber with sapphire discs from the incubator and place it on a prewarmed metal block.
    - Assemble each sandwich as shown in Figure 2D–2I. For this, place the sample holder under a stereomicroscope (Figure 2D).
    - Place an aluminum planchette with the 40 μm cavity facing up inside the holder (Figure 2E) and fill the cavity with 1 μL of the pre-warmed DMEM high-pressure freezing solution (Figure 2F).
    - Take one sapphire disc from the 3D-printed chamber and remove it slowly from the chamber.
    - Dip the sapphire disc with cells in the pre-warmed DMEM high-pressure freezing solution (Figure 2G).
    - Flip the sapphire disc so that the attached cells face downward. Then, gently place a sapphire disc on top of an aluminum carrier (Figure 2H). Check that air bubbles are not trapped between the aluminum carrier and the sapphire disc, as this will result in bad cryo-immobilization and eventually a breaking of the sapphire disc during freezing.
    - Immediately proceed by closing the sample holder (Figure 2I) and freeze the specimen using the high-pressure freezing machine.
    - Retrieve the *sandwich* of the aluminum planchette and the sapphire disc with the cryo-immobilized cells by opening the sample holder under liquid nitrogen. Press with a cold tweezer from the back and place the sapphire discs inside a cryo-tube. Be careful not to lift out the specimen from the liquid nitrogen bath during this procedure.
    - Store all cryo-immobilized specimens under liquid nitrogen until further use.
- **Freeze-substitution and embedding in Epon/Araldite**
  After cryo-immobilization, it is necessary to perform freeze-substitution of the cells (McDonald and Müller-Reichert, 2002; Studer et al., 2008). This process allows for a slow exchange of cellular water for acetone and later for epoxy resin (Epon/Araldite). The addition of osmium tetroxide preserves the cellular ultrastructure for subsequent transmission electron microscopy (TEM) (Mollenhauer, 1964). This process also introduces heavy metals (osmium tetroxide and uranyl acetate) to the sample, thus providing contrast for electron microscopy. We also add methylene blue to the freeze substitution *cocktail* to selectively stain chromosomes for imaging of the resin-embedded samples by light microscopy (Kiewisz et al., 2021). This step allows for the selection and staging of mitotic cells before TEM.
  1. Freeze-substitution of cryo-immobilized specimens:
    - Prepare saturated methylene blue solution in acetone by adding 5 g of methylene blue powder into 60 mL of anhydrous, glass-distilled acetone at room temperature and mix it well.
    - Store the solution in a 50 mL Falcon tube and leave it at room temperature for 2 h.
    - After that time, store the solution at -80 °C overnight.
    - After removing the solution from the -80 °C freezer, quickly filter the dye-saturated acetone solution with a syringe filter.
    - Prepare a freeze-solution cocktail by adding 1% (w/v) osmium tetroxide and 0.1% (w/v) uranyl acetate to the dye-saturated acetone. Store 1 mL aliquots of this freeze-substitution solution in 2 mL cryo-vials in liquid nitrogen until further use.
    - Open the high-pressure frozen sandwiches under liquid nitrogen and transfer the sapphire discs to the cryo-vials containing the AFS cocktail.
    - Perform freeze-substitution in an automated freeze-substitution machine. Keep samples at -90 °C for 1 h, before warming up to -30 °C with increments of 5 °C/h. Then, keep samples at -30 °C for 5 h, and again warm up to 0 °C in steps of 5 °C/h. At 0 °C, remove the sapphire discs from the cocktail (samples should not remain at 0 °C for more than one hour to avoid overstaining).
  2. Infiltration with Epon/Araldite (resin) and specimen embedding:
    - Wash the samples one time with pure anhydrous acetone at room temperature inside the cryo-vials (this will warm up a sample to room temperature in one rinse step) and then transfer the sapphire discs to a glass Petri dish filled with anhydrous acetone.
    - Place the Petri dish under a binocular microscope and arrange each sapphire disc with cells facing up. To determine the side of the disc with the attached cryo-immobilized cells, the sapphire discs are inspected for cellular contents under the dissection microscope while remaining immersed in acetone.
    - Transfer each sapphire disc to round flow-through rings within a plastic reagent bath (Leica), designed for 3 mm sapphire discs, with the cells facing up. Pre-fill the molds to a low level already with a 1:3 (v/v) resin/acetone mixture to prevent the sapphire discs from drying out. After all sapphire discs have been transferred to the plastic molds, check again under a dissection scope that all are properly placed in the indentations at the bottom of the molds. Then, fill up the mold to at least three-quarters with the acetone/resin mixture.
    - Infiltrate the samples with Epon/Araldite resin at room temperature with increasing concentrations of resin/acetone (v/v). Start with 1:3, then 1:1, and then 3:1 (1 h for each step). The exchange of the resin solutions should be performed very slowly to prevent the detachment and loss of cells from the surface of the sapphire discs and to avoid movement of the discs.
    - As a final step, fill the block molds with pure Epon/Araldite resin, and incubate the samples at room temperature overnight. It is important to completely fill the molds to avoid a meniscus of the resin within the individual mold subunits. This will cause a lensing effect of the transmitted light in the light microscope that is used to screen the blocks for cells of interest during the screening step.
    - Polymerize samples at 60 °C for at least 48 h in an oven.
  3. Release of resin-embedded cells from sapphire discs:
    - Cut the plastic molds open with a scalpel or a mechanical multitool (Dremel Fortiflex). Break the sample blocks out of the molds using a desmotome VT1 (a tool with a sharp arrow-shaped tip) as a chisel and a hammer. Under an ultramicrotome, carefully remove the resin around and on top of the sapphire discs using a single-edge razor blade. This needs to be done with great care to avoid breakage of the sapphire disc edges (Figure 4).
      **Figure 4.**
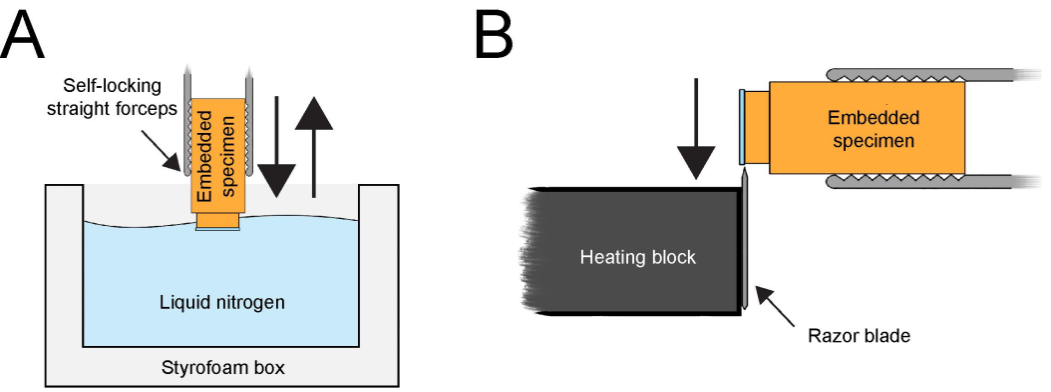
Removal of a sapphire disc from the Epon/Araldite block after resin polymerization. (A) Resin block with attached sapphire disc locked in self-locking forceps and dipped into liquid nitrogen. Only the sapphire disc should be submerged in liquid nitrogen for a few seconds. (B) Using a heating block to remove the sapphire disc from the cold resin block. After a few seconds, when the ice is starting to form on the surface of the cold sapphire disc, the edge of the sapphire disc is pressed to the heated razor blade of the heating block. This will leave the embedded cells (not shown here) in the resin. This figure was reproduced with permission from the book chapter: “High-throughput screening of mitotic mammalian cells for electron microscopy using classic histological dyes”, Methods in Cell Biology (162), 151–170 (2021), Copyright Elsevier [Kiewisz et al. (2021)].
    - Lock each sample in the self-locking straight forceps (artery clamp). Submerge only the top part of the resin block containing the sapphire disc into liquid nitrogen for a few seconds (Figure 4A). Avoid submerging the block too deep into the liquid nitrogen as this could break the resin block.
    - Remove the discs by gently pressing the edge of the discs to a heated razor blade (Figure 4B). The sapphire discs should self-release from the surface of the specimen without using extensive force, due to the different shrinkage and expansion of the resin and the sapphire disc exposure to the extreme temperature. If this method fails after 2–3 attempts of dipping, go back to step C3a and make sure that the resin from the sides of the sapphire discs is removed completely. This should allow a smooth removal of the sapphire disc from the resin block.
- **Pre-screening of samples and serial-sectioning of a selected specimen**
  Cells are located at the surface of the resin blocks (Kiewisz et al., 2021). Therefore, it is easy to pre-screen cells for subsequent ultramicrotomy. Although a standard light microscope can be used for this purpose, rare stages like prometaphase or early anaphase are hard to spot with certainty when relying solely on the different refractive indices within the resin. A fluorescence microscopy approach can be applied to obtain high-resolution image stacks that allow for identifying the desired stages. To achieve this, methylene blue or other histological dyes (Kiewisz et al., 2021) are added during freeze-substitution to specifically stain the chromosomes. This approach enables the screening of cells of interest before sectioning the specimens.
  1. Using an upright transmitted light microscope, screen the surface of the resin block to locate cells that are rounded and show any mitotic hallmarks, e.g., condensed chromosomes aligned at the metaphase plate. Image the area of interest with a microscope camera or through the eyepiece with a smartphone. This step is necessary as the transmitted light microscope can only be used to observe cells that are relatively shallow.
  2. For a better relocation of cells of interest, it is advised to image the area at different magnifications (Figure 5B and 5C).
    **Figure 5.**
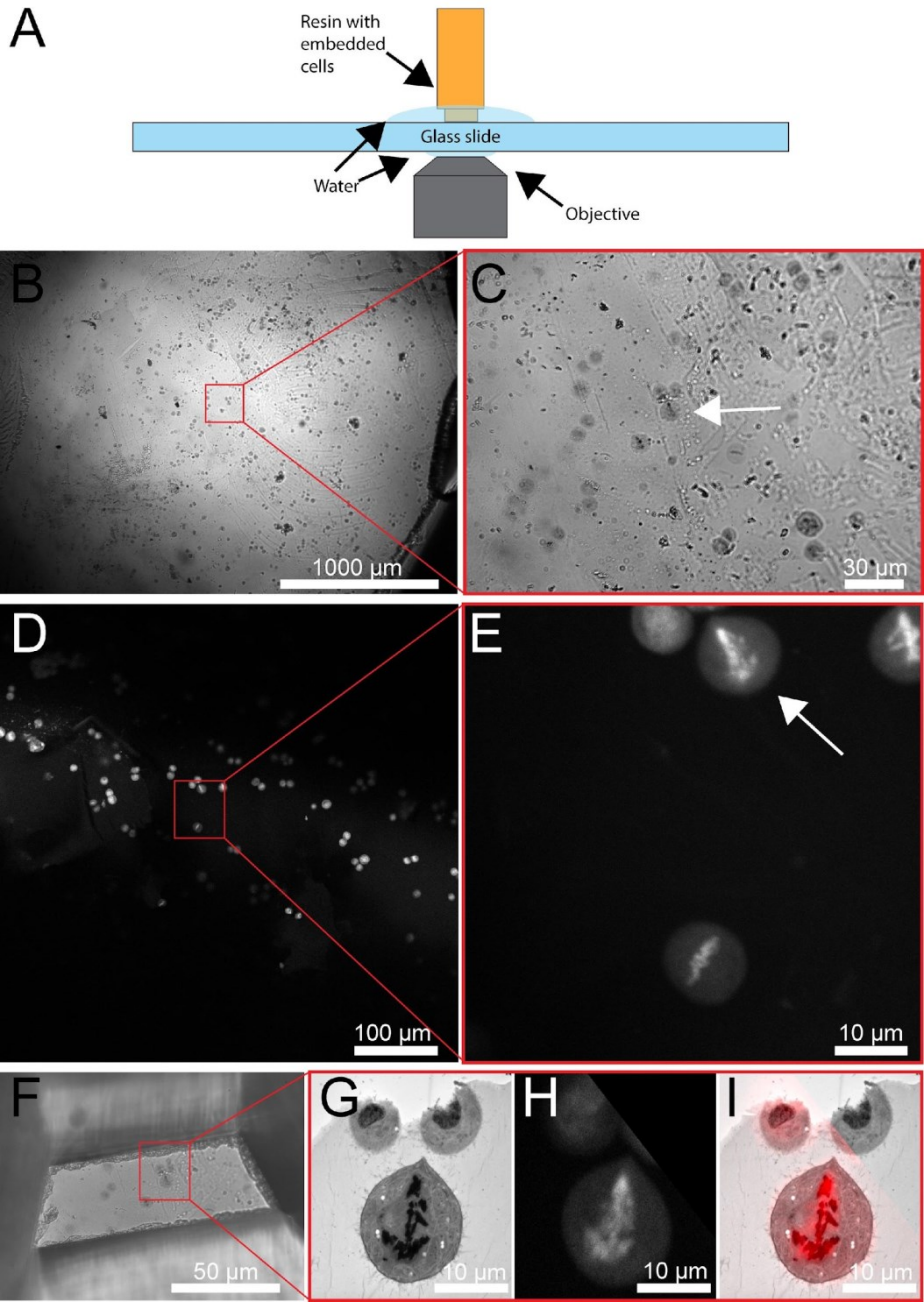
Pre-screening of resin-embedded HeLa cells using methylene blue. (A) Cartoon showing assembly of resin embedded cells on the microscope stage. (B) Overview image of the region of interest (red box) using an upright light microscope. (C) Higher-magnification image of the region of interest is shown in B. The cell of interest is indicated by a white arrow. (D) Low-magnification fluorescence image of resin-embedded HeLa cells stained with methylene blue. The region of interest is indicated by a red box and corresponds to the area of interest as shown in B. (E) Higher-magnification image of the region of interest as shown in D. The cell of interest is indicated by a white arrow. (F) Razor blade-trimmed resin block containing the cell of interest. (G) Electron micrograph of a 300 nm section of the selected cell. (H) Corresponding fluorescence light microscopic image of the cell of interest. (I) Overlay of the fluorescence light and electron microscopic images, as displayed in G and H. This figure was reproduced and modified with permission from the book chapter: “High-throughput screening of mitotic mammalian cells for electron microscopy using classic histological dyes”, Methods in Cell Biology (162), 151–170 (2021), Copyright Elsevier [Kiewisz et al. (2021)].
  3. Position a resin block with the specimen side facing the objective of a confocal microscope (such as the Leica SP5 or Leica SP8 Stellaris). It is advised to use an inverted microscope for this and place the resin block on top of a 24 mm × 60 mm coverslip. The block should be placed on the glass slide with the cells facing down and submerged in a droplet of water (Figure 5A). We found that using a 594 nm excitation laser and detecting the emitted light in the range of 620–730 nm yields the best results for methylene blue as a dye. Make sure to relocate areas of interest by identifying *landmarks* such as the distribution pattern of the embedded cells or any distinctive features (e.g., dirt).
  4. Collect images of the putative areas of interest and validate if the areas contain the cells of interest (Figure 5D–5E) by investigating the stage of the selected cells. At this point, damaged cells can be excluded from further processing.
  5. Re-localize a region of interest using either an upright brightfield microscope or the microscope attached to the ultramicrotome (Figure 6A–6C).
  6. Trim resin block surface for serial sectioning. We use razor blades for this step. Start with a rather large surface area (Figure 6D; Video 2).
  7. Again, re-localize the region of interest using an upright brightfield microscope (Figure 6E–6G).
  8. Repeat steps D6–D7 until the sample has been trimmed to the appropriate size (Figure 5F and Figure 6H).
  9. On an ultramicrotome, equipped with a diamond knife, overfill *the boat* of the diamond knife with water.
  10. Add 1–3 droplets of 70% ethanol.
  11. Remove some water from the boat with a syringe until a bright reflection is visible at the edge of the diamond.
  12. Move the trimmed specimen very carefully to the edge of the knife.
  13. Cut 300 nm sections with a speed of 1.6 mm/s. The cutting speed can vary by ± 0.3 mm/s, depending on the room temperature and humidity as well as the polymerization stage of the sample (Figure 6I–6J).
  14. Move ribbons of sections with hair glued to the tip of a toothpick. We prefer to use Dalmatian dog hairs for this procedure. However, any other non-sticky type of hair can be used for this.
  15. Collect ribbons of sections on Formvar-coated copper slot grids (Figure 6K).
    **Figure 6.**
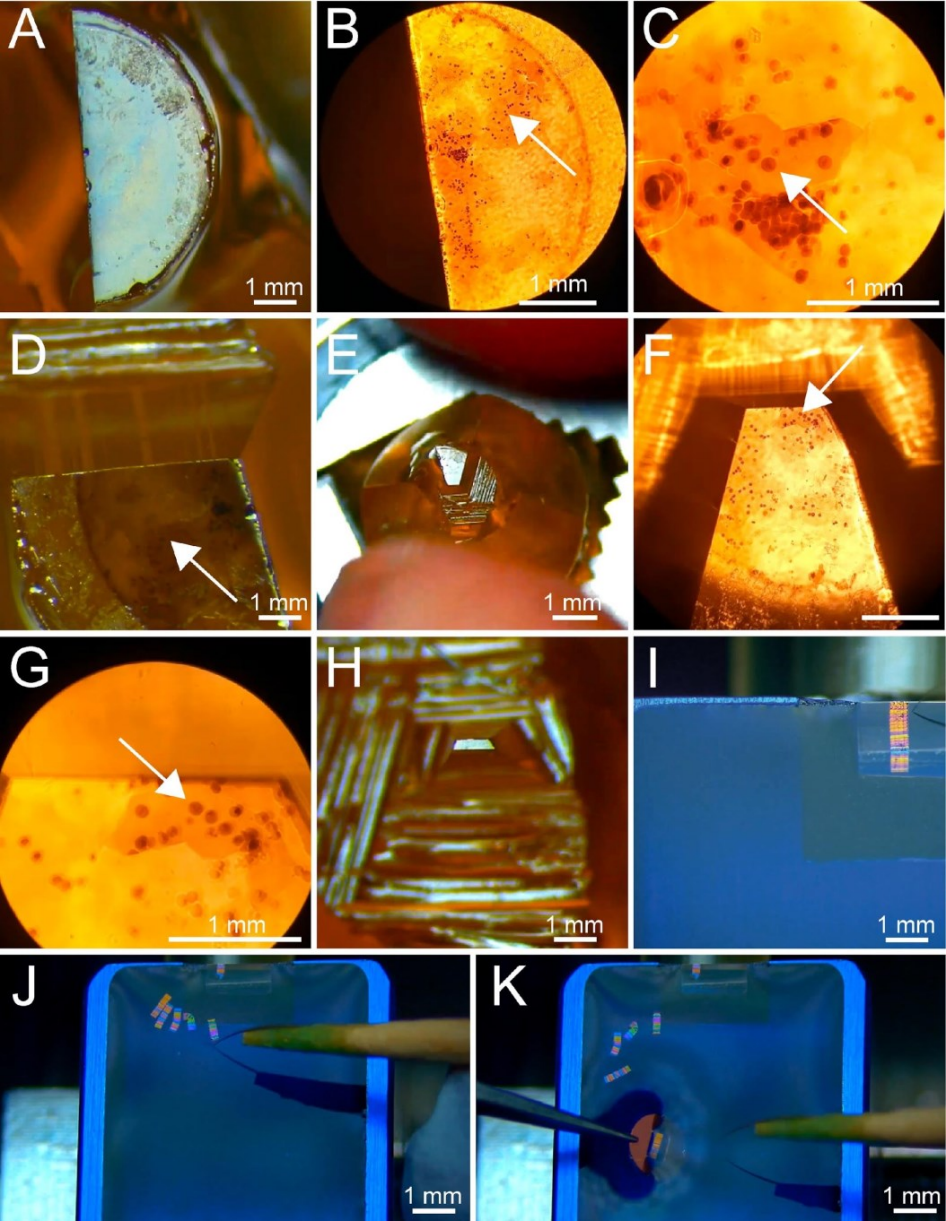
Serial sectioning of selected cells in resin-embedded samples. (A) Top view showing a partially trimmed resin block. Trimming of the block is achieved using a razor blade. (B) Top view of the same resin block as shown in A. The image was taken by using a stereomicroscope at 5× magnification. The selected cell is indicated by a white arrow. (C) Zoom-in view of the area of interest at 25× magnification as shown in B. The selected cell is indicated with a white arrow. (D) Rough trimming of the resin block around the area of interest. (E) Top view of the further rough trimmed area around the cell of interest as seen by using a binocular microscope. (F) Top view of the same resin block as shown in E imaged using a stereomicroscope at 5× magnification. The arrow indicates the cell of interest. (G) Zoomed-in view of the area of interest at 25× magnification with the indicated cell of interest white arrow as shown in F. (H) The top view of the fully trimmed trapezoid-shaped block face of the resin sample. (I) Top view onto *the boat* of a diamond knife showing a ribbon of serial sections attached to the knife edge. (J) Top view onto the boat of a diamond knife showing five ribbons of serial sections. At this stage, it is crucial to keep the correct sequence of the ribbons. (K) Same view as given in J to illustrate how a single ribbon is collected on a Formvar-coated copper slot grid (see also Video 2).
    **Video 2.**
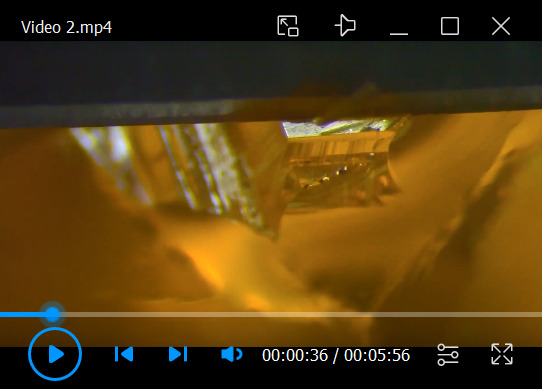
Video tutorial showing how to perform serial-section ultramicrotomy. Trimming of a resin block containing a selected cell is achieved by using a razor blade. This is followed by serial sectioning of the trimmed area using a diamond knife. This video showing serial sectioning is speeded up. This video corresponds to Figure 6.
  16. Post-stain sections with uranyl acetate and lead citrate and image sections by using a TEM (Figure 5G).
  17. Add fiducial markers to the sections by immersing them into a droplet of colloidal gold (Ø 20 nm) solution for 1 min. Then, blot excessive fluid with filter paper and air dry the grid for 1 min. Wash grids for 1 min in double-distilled water and let them air dry before storing them in a grid box.
  18. Inspect the serial sections on the grids by using a TEM operated at 80 kV. This process allows to inspect and validate the selected cells by overlaying the electron micrograph with the fluorescence image previously taken (see step D3; Figure 5G–5I).
  19. After this control step, image-select cells with a TEM operated at 300 kV and equipped with a tilting stage (Mastronarde, 1997) with SerialEM software. For a detailed tutorial for acquiring tilt series tomography with this software, we refer to the online tutorial (https://bio3d.colorado.edu/SerialEM/).
    - Load the grid with the serial sections into a tomography sample holder and insert it into the TEM.
    - Open SerialEM, create a new map file, and mark the position of a cell of interest on each section on the grid. Make sure to adjust the Eucentric height at all positions as this will speed up the acquisition process.
    - Using the SerialEM *pre-exposure* feature, expose each selected position on each section to 2,000 e-/Å^2^ at a 60° tilt angle for 5 min. This *baking* process allows the resin section to shrink to a stable level before the image acquisition is started.
    - Set up serial acquisition in SerialEM to acquire a tilt series for each region at ± 60° with 1° increments at 4,700× magnification (a-axis tilt series).
    - After all acquisitions are finished, rotate the grid by 90° and repeat steps D19b and D19d to record the b-axis tilt series.
- **Automatic reconstruction of electron tomogram and microtubule segmentation**
  Following the tomographic acquisition, each tilt series file has to be reconstructed to obtain an in-silico 3D volume. This process can be time consuming, which is why we chose to fully automate this process. Tomogram processing is carried out with the IMOD software package. The IMOD software package is a set of image processing, modeling, and display programs for tomographic reconstruction and 3D reconstruction of EM serial sections. Automation scripts and instructions for an automatic tomographic reconstruction with IMOD are available on GitHub (https://github.com/RRobert92/Assit_Automatic_Tomogram_Flattening_IMOD). For full documentation and a tutorial on how to use IMOD, we refer the reader to the IMOD tutorial (https://bio3d.colorado.edu/imod/doc/etomoTutorial.html).
  1. Automatic tomogram reconstruction:
    - To start an automatic tomogram reconstruction, open the etomo package from the IMOD software and follow step-by-step instructions from File S3.
    - Open the batch processing mode.
    - Load the automatic reconstruction setting available on GitHub (https://github.com/RRobert92/Assit_Automatic_Tomogram_Flattening_IMOD/blob/main/Kiewisz_Imod_batch_reconstruction.adoc).
    - Select raw data files (tilt series). Each pair of tomogram files (a- and b-axis) should be stored in a separate folder.
    - Choose a device (CPU, GPU, or CPU + GPU) on which the tomogram will be processed.
    - After pressing *Run*, the tomographic reconstructions will be calculated and stored on the hard drive. If the message shows *completed*, proceed to step E1g. If the message shows *failed*, one needs to check the step on which the algorithm aborted the reconstruction and fix it manually.
    - Post-process reconstructed tomograms by trimming the histogram and rotating the volume around the x-axis.
    - Run an automatic flattening tool from GitHub (https://github.com/Robert92/Assit_Automatic_Tomogram_Flattening_IMOD/blob/main/AAFT.bat) to flatten each tomogram.
    - Use the *new stack* command from IMOD in the terminal to manually remove z-planes from the 3D stack that do not contain image information.
  2. Semi-automatic microtubule segmentation: as previously published (Redemann et al., 2014), microtubules are automatically segmented using the commercial Amira or AmiraZIBEdition software package. The Amira software package is a set of image processing and visualization tools that allows for precise and intuitive image segmentation of big data. A tutorial of this process is shown in Figure 7 and File S4. For a full tutorial on the filament segmentation see https://assets.thermofisher.com/TFS-Assets/MSD/Product-Guides/user-guide-amira-software.pdf.
    **Figure 7.**
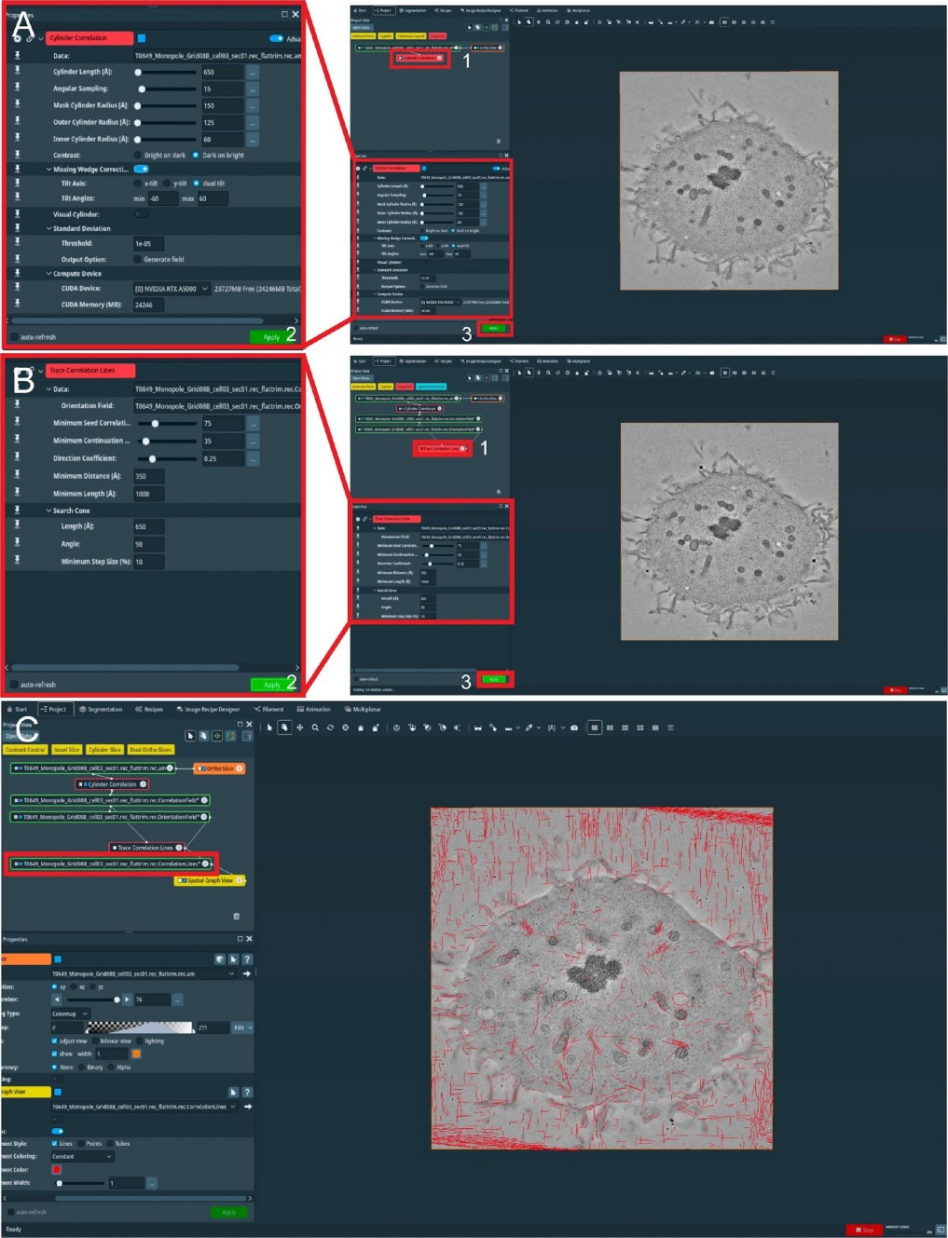
Semi-automatic segmentation of microtubules in whole spindles obtained from electron tomography of serial sections (300 nm). (A) View of the Amira graphical user interface (GUI) showing the steps to initialize semi-automatic microtubule prediction by computing correlation and orientation fields. To initialize the procedure, select the *Cylinder Correlation* tool (shown in red box 1, right panel), and select parameters for microtubule template matching (shown in the left panel, red box 2). To start the calculation, press the *Apply* button indicated with red box 3. (B) GUI showing steps to track microtubules from template matching, generated in A. To initialize microtubule tracing from the generated template, use the *Trace Correlation Lines* tool (red box 1, right panel) and select parameters for microtubule tracing (shown in the left panel, red box 2). To start tracing, press the *Apply* button indicated with red box 3. (C) GUI showing the final spatial graph file containing coordinates for the detected microtubule tracks. The red box indicates all traced microtubule filaments stored in .am Amira spatial graph file format.
    - Load reconstructed tomogram volume into the Amira software (Figure 7).
    - Select the *Cylinder Correlation* tool (Figure 7A, red box 1) and select the setting as shown in Figure 7A (red box 2).
    - Press the green button *Apply* to start the calculation (Figure 7A, red box 3).
    - After the calculation is finished, two new files are produced (Figure 7B).
    - Select the *Trace Correlation Lines* tool. Then, select the two new files as inputs and use the setting as shown in Figure 7B (red box 1–2).
    - Press the green button *Apply* to start the calculation (Figure 7B, red box 3). At the end of the calculation, a new file will be created called a *spatial graph* containing the predicted microtubule tracks (Figure 7C). Save the file locally on the hard drive.
    - Be aware that Amira allows the generation of so-called *Recipes*. Within these Recipes, one could save the configuration of certain modules like *Cylinder Correlation* and *Trace Correlation Lines*. Therefore, one has only to apply the recipe to the raw tomogram to obtain the *spatial graph* as a resulting output, which saves time and prevents human errors in configuring the parameters of the modules. We recommend using the properly configured *Recipes* when segmenting microtubules if the tomogram quality is not strongly varying.
  3. Correction of microtubule tracks predicted with the Amira software. During the prediction of microtubule tracks from electron tomograms, several errors can occur as false-positive or false-negative microtubule tracks. This correction needs to be done manually and therefore can take up to several hours or a full working day per single tomogram (Figure 8A–8B). Each acquired tomogram needs to be corrected.
    **Figure 8.**
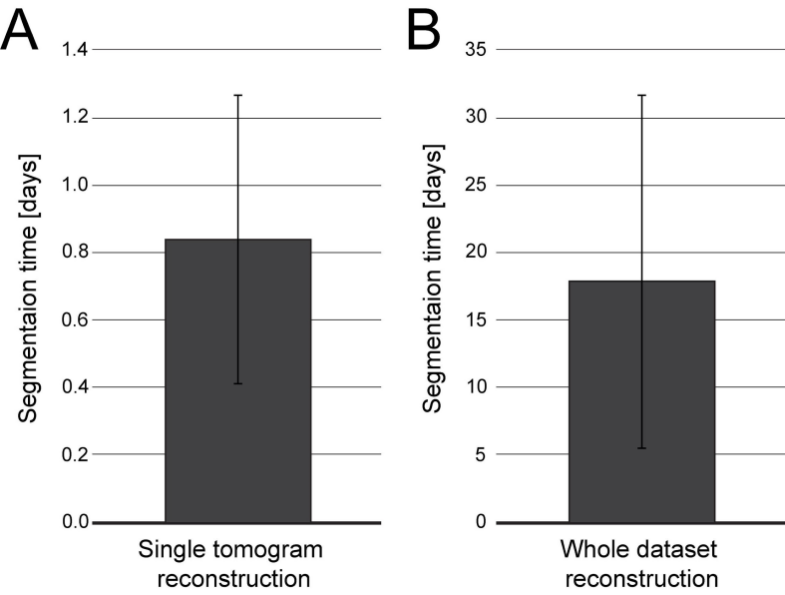
Plot indicating the time needed for microtubule segmentation using the Amira software including semi-automatic segmentation and manual correction by experienced users. Segmentation time is shown in full days referring to 24 h time intervals. (A) Bar plot showing the segmentation time per single tomogram (n = 195). (B) Bar plot showing the segmentation time per entire data set (n = 14). Each data set is composed of 8–37 tomograms.
    - For correction, open the filament tab in the Amira software (Figure 9, red box).
      **Figure 9.**
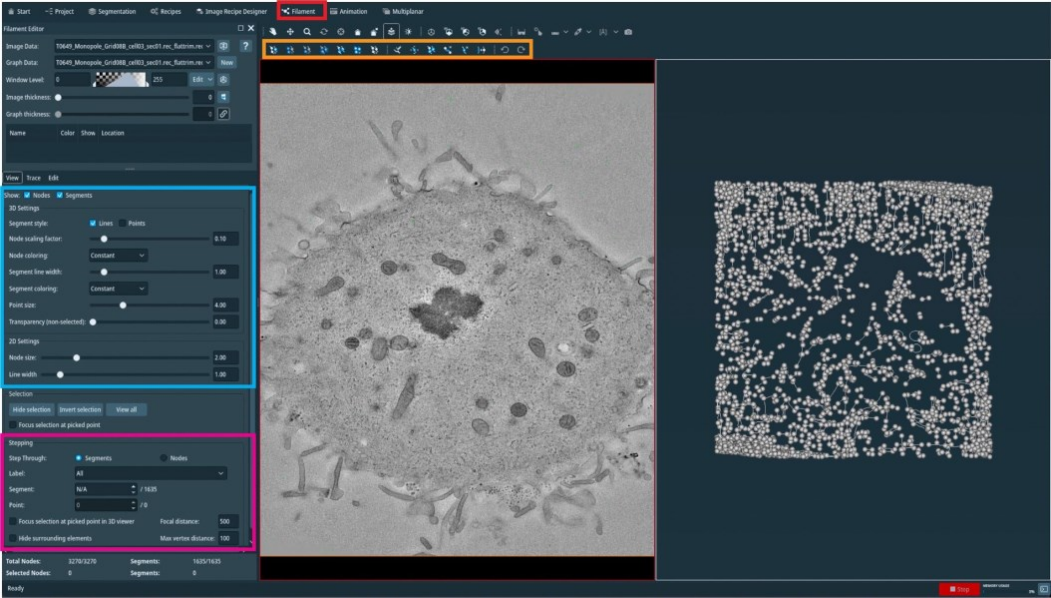
Manual correction of semi-automatically segmented microtubules using the AmiraZIBEdition software. GUI showing an overview of the filament editor tool (red box, top). The orange box indicates the different tools that can be used to correct the segmentation, the blue box shows visualization parameters, and the pink box indicates the stepping tool used to step through all individual microtubules.
    - In an open new window, one can now see, side-by-side, the tomogram and all predicted microtubule tracks.
    - On the top of the open window (Figure 9, orange box), one can see all the editing tools. By highlighting with the mouse cursor, one can read the descriptions and the keyboard shortcuts associated with them.
    - On the top-left side of the open window (Figure 9, blue box), one can see the visualization setting of the spatial graph that controls the thickness of displayed lines.
    - On the bottom-right side of the open window (Figure 9, magenta box), one can see the editing tool where one can switch between segmented microtubules.
    - Correct the spatial graph by removing all false-positive segments and adding the missing (false-negative) microtubule tracks.
    - Remove the false-positive microtubules (Figure 10A) by selecting them in the *segment* box menu (Figure 9, magenta box) and pressing D (for delete) on the keyboard. To add a new microtubule track, firstly press C and then T, and select the center of the microtubule by clicking on it within the left image viewer. This will create the first node. To extend the newly created microtubule track, press N, and while pressing the control key, continue to select and click in the center of the microtubule to draw a line until reaching the end of it.
      **Figure 10.**
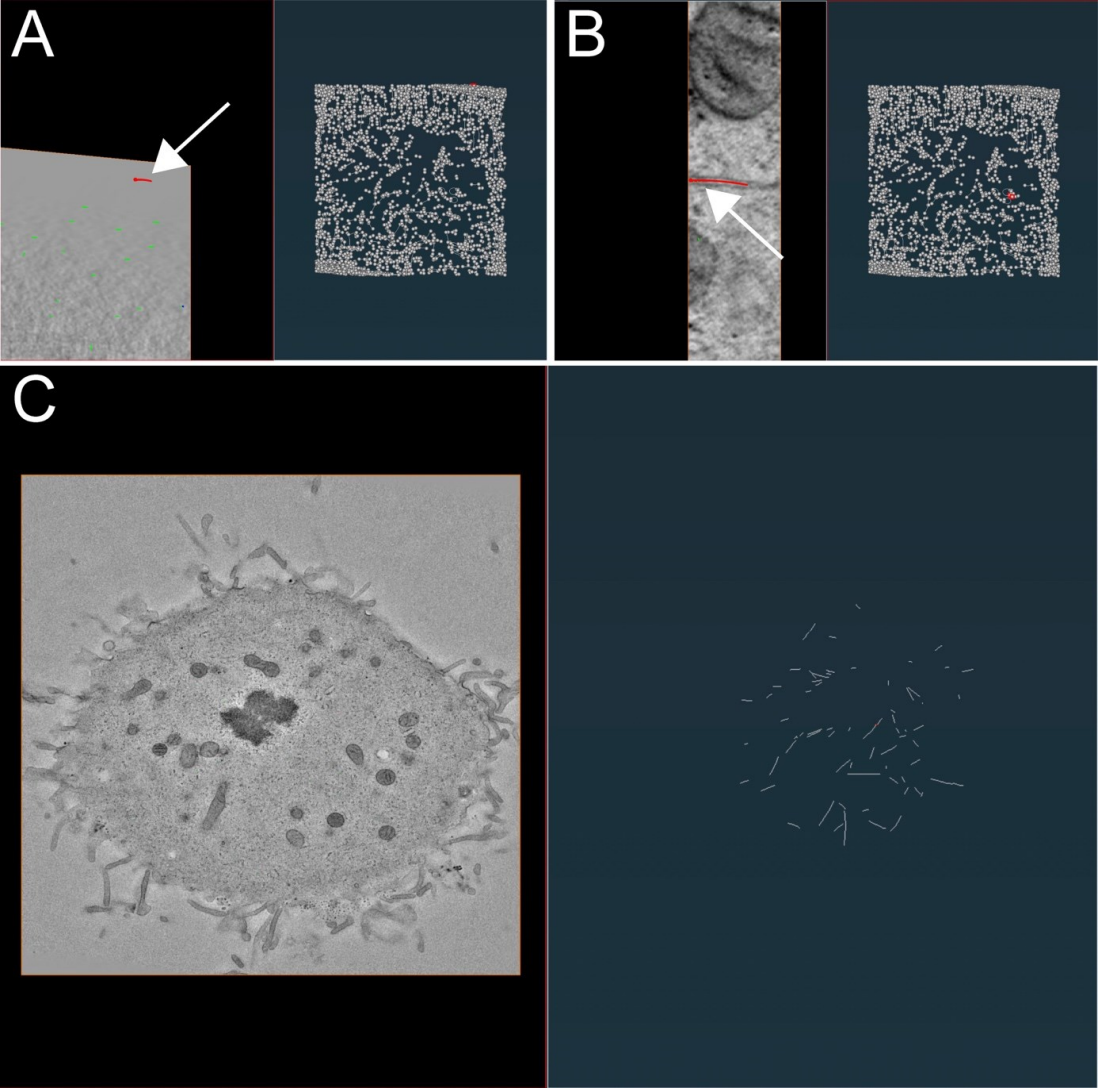
Illustration of microtubule tracing correction using the AmiraZIBEdition software package as shown in Figure 9. (A) Example of false-positive filament annotation caused by the application of the semi-automatic segmentation approach. The left panel shows a cross-section view of a tomogram with selected microtubules highlighted in red and white arrow. The right panel shows an overview of all traced microtubules with selected microtubules highlighted in red and white arrow. (B) View from the AmiraZIBEdition software as shown in A. Example of a correctly tracked microtubule. (C) View from the AmiraZIBEdition software as shown in A. Example of manually and fully corrected filament annotation.
    - Check if the microtubule track is correct and if the ends of the segment are positioned at the ends of the microtubule in the image. One can do that by moving through the Z-planes with the mouse wheel up and down.
    - If the selected microtubule track looks correct, one can simply skip to the next one (Figure 10B) until all microtubules have been corrected and all false positive segmentations have been deleted (Figure 10C).
  4. Stitching of segmented serial sections. The final step of microtubule segmentation is the stitching of all serial sections together to one tomographic volume with all microtubules stitched together to one spatial graph. For this, we used a research version of Amira called AmiraZIBEdition comprising the *SerialSectionStack* tool (Lindow et al., 2021). This tool is also available on GitHub (https://github.com/zibamira/SerialSectionAligner or https://www.zib.de/software/serial-section-aligner) as an extension for Amira v2020.3 or higher.
    - Open Amira with the installed extension or AmiraZIBEdition and create the *SerialSectionStack* tool (Figure 11A, red box 1).
      **Figure 11.**
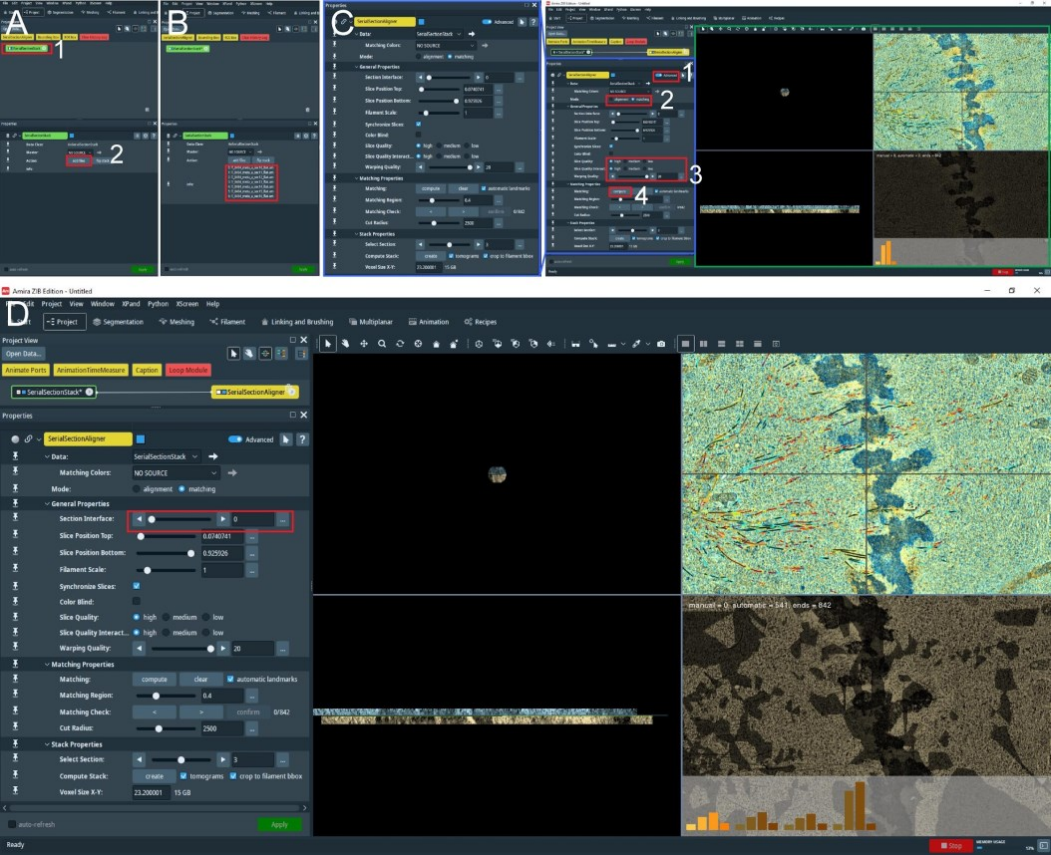
Stitching of serial sections by using the AmiraZIBEdition *SerialSectionStack*. (A) The main view of the AmiraZIBEdition software GUI. The red box number 1 indicates the data management window with the open *SerialSectionStack* tool. The red box number 2 indicates the button used to load all serial section data for stitching. (B) GUI view as shown in A. Loaded datasets in the correct order are indicated in the red box. (C) View of AmiraZIBEdition software GUI showing open *SerialSectionAligner* tool setting window. The left panel shows the zoomed area as shown in the blue box in the right panel. The numbered red boxes show the sequence of tasks that have to be done to initialize the *SerialSectionAligner* tool: No. 1, *Advanced* button; No. 2, *Switch to matching mode*; No. 3, *Switch slice quality to high and the warping quality to the maximum*; and No. 4, *Compute the alignment of the transformation matrix for the first section by pressing Compute*. The green box shows the alignment view window, allowing the user to observe and improve the alignment. (D) To compute the alignment transformation matrix for the next section, the user needs to switch to the next border using the buttons indicated in the red box.
    - From the project tab (bottom left corner, Figure 11A, red box 2) select *add files* and select all tomographic volumes with corresponding spatial graph fields. The files should be named with consecutive numbering e.g., Tomogram_1.am, Tomogram_sg_1.am; Tomogram_2.am, Tomogram_sg_2.am (Figure 11B, red box), and they should be all in the same folder.
    - With the right mouse button, press the *SerialSectionStack* tool and open the *SerialSectionAligner* tool. This will update the *properties* tab view (Figure 11C, right panel, green box).
      Initialize this tool by pressing *Advanced* (Figure 11C, red box 1) and switch to matching mode (Figure 11C, red box 2). Enable a high-quality display for tomographic slices and increase the warping quality to the maximum (Figure 11C, red box 3).
    - Next, press *compute* (Figure 11C, red box 4). This will calculate the alignment transformation matrix for the first section based on matching microtubules from the first and the second section. When finished, the computation view window (Figure 11C, right panel, green box) will update to show the aligned two consecutive sections. Repeat this step a few times until the histograms (shown in the bottom right corner) do not change anymore.
    - Repeat step E4e for each section by switching to the next section interface (Figure 11D, red box) and pressing *compute*.
    - When the computation of the alignment matrix for all sections based on microtubule continuity between sections is finished, press *create* at the bottom of the *properties* view. One can optionally deselect *crop to filament*. This option, when enabled, allows the tool to crop tomogram volume to the spatial graph size and produce a smaller tomogram volume (Figure 12A).
    - At the end of the calculation, a user obtains two files (Figure 12B, red box 1–2): the Amira spatial graph with all stitched microtubules and a 3D volume of the stitched serial sections (Figure 12C–12D).
      **Figure 12.**
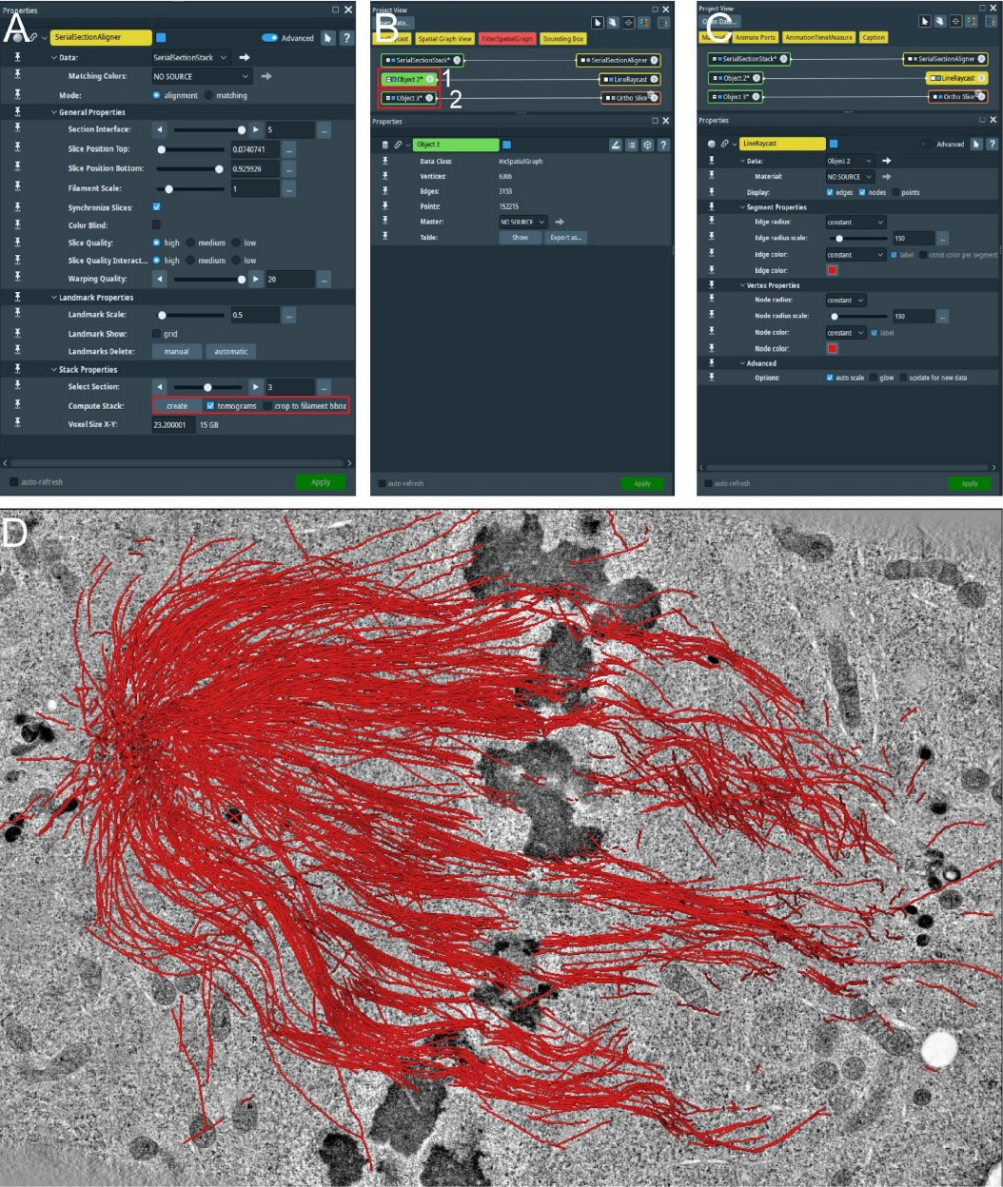
Correction of stitched microtubules from all serial sections using AmiraZIBEdition *SerialSectionStack*. (A) After repeating the computation for all borders in between sections (see Figure 11D), the user can compute the stacked volume for all aligned sections using the computed alignment transformation matrix by pressing *Create* (red box). (B) At the end of the step shown in A, two new files are generated. The stitch tomogram volume (red box number 1) and corresponding file with all stitched microtubules (red box number 2). The file as indicated by the red button is the Amira Spatial Graph file that contains tracks for microtubules stitched from all serial sections. (C) View from AmiraZIBEdition as shown in A, indicating visualization setting. (D) View from AmiraZIBEdition showing a tomographic slice and all stitched microtubules using the setting as shown in C.
    - As an important final step, check and correct each stitched microtubule. For this, repeat step E3 for the generated stitched files.

## Data analysis

- **Analyses**
  In this step, we present an Automatic Spatial Graph Analysis tool (ASGA) that was developed for rapid local and online analysis of segmented microtubule tracks (Figure 13A–13C; Video 3). ASGA is a fully automated package with an intuitive GUI. After loading the data, the users only have to select the type of analysis that should be performed on the microtubule tracks. The entire code is developed in R and hosted as an online service via R shiny package. In the following part, we present all available analyses in ASGA (links and GitHub: https://github.com/RRobert92/ASGA; Kiewisz and Müller-Reichert, 2022b).
  **Figure 13.**
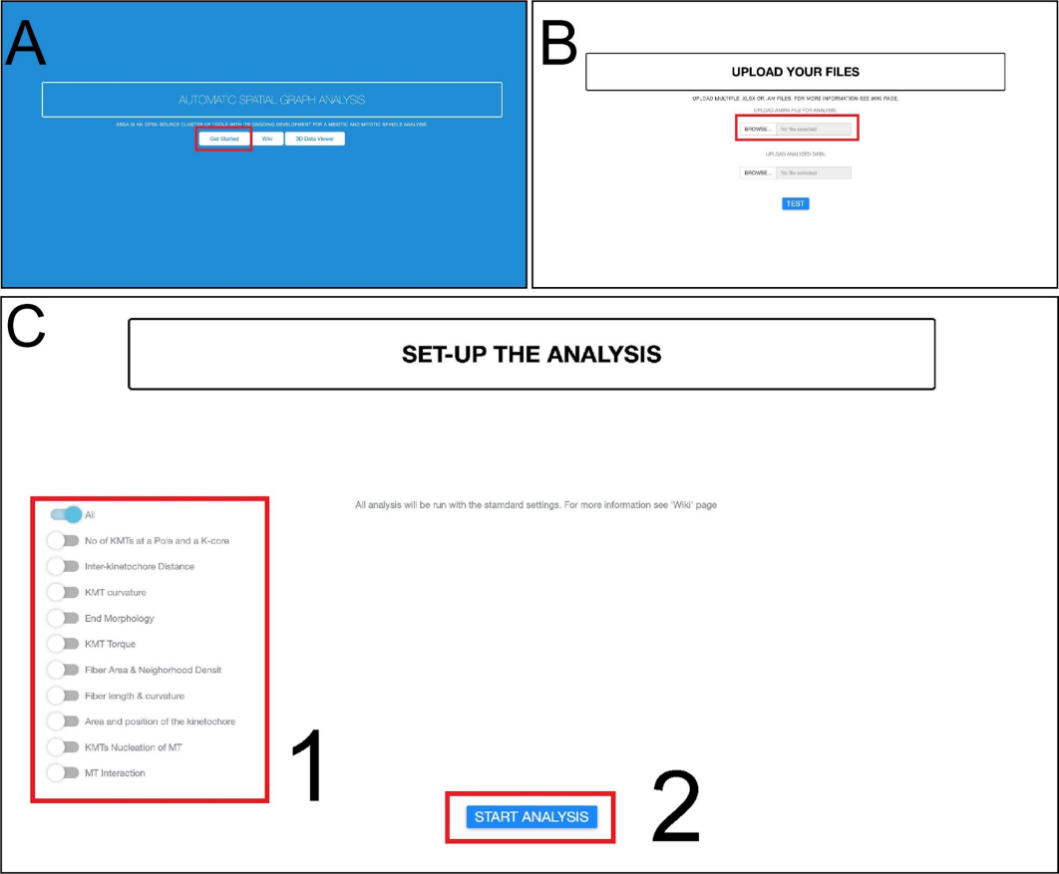
Workflow for fully automatic microtubule analysis with ASGA. (A) The home screen of the ASGA software. The red box indicates the button used to start the analysis. (B) Loading of data into the ASGA software. The red box shows a window where users can select either single or multiple datasets at once. (C) After the data compatibility has been checked, the ASGA software welcomes the user with a setup window. This window allows users to customize their analyses. A selection of the options is needed at this point. The red box number 1 allows one to select the type of analysis. The user can switch on or of specific types of analysis. The red box number 2 indicates a button to start the selected type of analysis (Kiewisz et al., 2022; see also Video 3).
  **Video 3.**
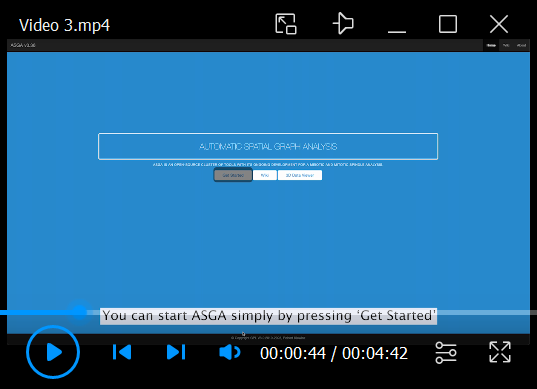
Tutorial video describing the usage of the ASGA software v0.36. This video corresponds to Figure 13.
  1. Data format: the ASGA workflow is operating on the ASCII Amira file format. This file can be exported from the AmiraZIBEdition package and contains information about each microtubule spline. In addition, for the analysis of the kinetochore microtubules (KMTs), the file should have separated microtubules into KMTs and non-KMTs. We have built in the ASGA workflow a file format error handler that checks each input data structure and gives clear information if any of the files is not compatible with ASGA and provides hints on how to fix it.
  2. Fiber and KMT length distribution: the length of each microtubule is calculated as shown in Eq. 1. Furthermore, length distribution is calculated as a function of all lengths (Eq. 1).
$$\sum_{i=1}^{n-1}L=\;d_{i,\;i+1}+\ldots +d_{n,\;n+1}$$
    Eq 1. Equation calculating the length of microtubule track (L) using 3D points data, where *i* indicates the point belonging to the microtubule track and *d_i,i+1_* indicates the 3D Euclidian distance between two consecutive points.
  3. Statistical analysis of KMT number: ASGA performs a statistical analysis of the KMT number and compares it against the kinetochore position, the sister k-fiber KMT number, and the distance between the sister k-fiber microtubule plus-ends.
  4. Outer-kinetochore distance and distribution: the distance between outer kinetochores is calculated as the distance between sister k-fiber plus-ends. A k-fiber plus-end is taken as a mean 3D position of all KMT plus-ends. K-fiber sisters can be indicated by the user in the spatial graph (see the New tools development paragraph) or automatically detected. Automat detection of k-fiber sisters is achieved by firstly estimating the kinetochore position with KMT plus-ends and then selecting a sister kinetochore that is facing the same direction.
  5. Fiber and KMT curvature: to calculate KMT or k-fiber total curvature, ASGA measures the local and total tortuosity (Eq. 2). Tortuosity is the measurement of the line deformation and can be between 1 and infinity, where 1 indicates a straight line. For example, 1.57 indicates the curvature of a half-circle. It is calculated as a ratio of the true microtubule length as calculated in Eq. 1 and the length of a straight line between its endpoints. This measurement can be used as a quantification of microtubule curvature indicating the deviation from a straight line. To calculate the tortuosity of a k-fiber, ASGA computes the center of a k-fiber as a mean of all microtubules in the fiber, and the k-fiber tortuosity is a measure based on this center of a k-fiber.
$$\tau =\frac{L}{l}$$
    Eq 2. Equation calculating microtubule tortuosity, where *L* indicates the total length of the microtubule calculated as in Eq1, and *l* indicates the microtubule length as a straight line between its endpoints.
  6. KMT twist: the local KMT twist is calculated along KMT in steps of 500 nm along the microtubule lattice. For each step, the twist is calculated as a clockwise or anti-clockwise angle of rotation (Eq. 3). The total twist is calculated as a sum of all local twists along k-fiber. For this, at each step, we calculate the mean twist value from each KMT present in the k-fiber, and the total k-fiber twist is given as a sum of all local twists along the k-fiber (Eq. 4). This means that, for example, if the k-fiber is randomly twisted, the total twist will be close to 0°, and if the k-fiber is in its entire length negatively twisted, the total twist of a k-fiber will be for example -25°.
$$Local\_Twist=arctan\frac{\vec{y_{i+1}}}{{\vec{x}}_{i}}\times \frac{180}{\pi }$$
    Eq 3. Equation calculating local microtubule twist between two consecutive points on microtubule tracks, where *x* and *y* indicate 2D vectors between the center of the k-fiber and KMT at a position *i* and *i+1.*
$$Total\_Twist=\sum_{i=1}^{n}{Local\_Twist}_{i}+{Local\_Twist}_{i+1}+\ldots +{Local\_Twist}_{n}\_$$
    Eq 4. Equation calculating summed microtubule twist along k-fiber, where *i* indicate the number of steps along which the local twist is calculated.
  7. K-fiber helicity: K-fiber helicity is calculated by firstly computing the k-fiber center and measuring the total twist of a k-fiber relative to the pole-to-pole axis. Helicity is then calculated as a k-fiber total twist over its length as shown in (Eq. 5).
$$Helicity=\frac{\sum_{i=1}^{n}{Local\_Twist}_{j}+{Local\_Twist}_{j+1}+\ldots +{Local\_Twist}_{m}}{L}$$
    Eq 5. Equation calculating k-fiber helicity, where the numerator indicates the total twist of a k-fiber based on a k-fiber center relative to the pole-to-pole axis, and *i* is the fiber length calculated with Eq1.
  8. Fiber area: to calculate the fiber cross-section area, ASGA uses the alpha shape algorithm. This algorithm allows one to fit a convex hull polygon into the cross-section of the k-fiber. This approach allows for precise retrieval of the cross-sectional area of the k-fiber. The fiber area is calculated locally every 500 nm, or as the total fiber area given as a mean value of all local areas.
  9. KMT neighborhood density: to calculate the local density of the KMTs in a k-fiber, the ASGA software computes the local fiber area at the specified position. The density is then derived as the number of KMTs divided by fiber area at a given position (Eq. 6).
$$Density=\frac{n}{{Fiber\_area}_{m}}$$
    Eq 6. Equation calculating k-fiber local density, where *n* indicates the number of KMTs at a given position *m*.
  10. KMT branching: to detect branching of microtubules, ASGA computes the distance matrix between each KMT minus-end and every microtubule spline (Eq. 7). Next, for each minus-end, ASGA selects the closest microtubule spline. Finally, KMT branching from either microtubules or KMTs is detected by selecting KMT minus-ends, which are within a specified user distance (default: 25 nm) to the closest microtubule spline. ASGA gives out a list of KMT branches with KMT ID, microtubule ID from which KMTs branched, and the distance of KMT minus-end to the microtubule spline.
$$D_{j}\;(\left|P_{i}-P_{j}\right|)\;$$
    Eq 7. Equation calculating the distance between the KMT end and every other microtubule spline, where *n* is the number of microtubules in a system, *i* indicates KMT 3D end position, and *j* indicates microtubule spline.
  11. Global microtubule–microtubule interactions: to identify every microtubule–microtubule interaction, ASGA computes a global distance matrix. In short, ASGA computes the distance of each microtubule to each other microtubule within a given threshold distance (default: 100 nm). Further, ASGA gives information about microtubule IDs for which the interaction was observed, for the position on the microtubule where the interaction occurred, and for the length of interaction.
- **New tools development**
  The ASGA raw code compiled packages for Windows, MacOS, or Linux is available on GitHub (Kiewisz and Müller-Reichert, 2022b; https://github.com/Robert92/ASGA). The online version of the platform is available on GitHub.
  1. ASGA standardized data input. As with any other software, ASGA requires standardized data input. ASGA requires the input data to be in the form of an Amira Spatial Graph file format saved in the ASCII format. ASGA recognizes KMTs by detecting the KMT label; other microtubules are recognized as non-KMTs. Optionally, the user can specify extra labels, thus classifying which KMTs belong to which k-fiber. To ensure accurate computation of microtubule positions in relation to the spindle axis, it is necessary to re-orient the spindle such that both spindle poles marking the pole-to-pole axis have the same x-coordinate in the spatial graph. Furthermore, for increased accuracy in the analysis, the spatial graph should be re-sampled to a uniform point distance of at least 20 nm. The ASGA 3DViewer expects as input either CSV or XSLX file formats with all ASGA analyses as well as the corresponding Amira file spatial graph.
  2. Spatial graph pre-processing and new tool development. ASGA performs data initialization during the loading of a spatial graph, which further standardizes the input data for all other tools to operate on. Each spatial graph is assigned an ID number, which the developer can refer to when computing multiple spatial graphs one by one. Additionally, each spatial graph is divided into three classes: non-KMTs, KMTs, and k-fibers. The optional k-fiber class is created if the spatial graph contains information for each KMT about its association with a particular k-fiber. To each microtubule, a unique ID is assigned. Developers can retrieve either individual microtubules or a set of microtubules belonging to a k-fiber by calling these IDs. By calling a specific microtubule ID, the developer retrieves access to a sorted indexed list of XYZ coordinates, where the first and last coordinate is associated with the ends of each microtubule. In addition, a developer can use the indexed list to mark only a specific area of a microtubule.
    As for the code of conduct, developers are advised to develop a function that operates on either an individual or a subset of microtubules and then wrap this function over the desired classes. This approach may not always be optimized for speed. However, it allows new developers not familiar with the code to easily create new tools that can be rapidly implemented and deployed.
- **Visualization**
  The ASGA-3DViewer raw code is available on GitHub (https://github.com/RRobert92/ASGA_3DViewer; Kiewisz and Müller-Reichert, 2022a). The online version of this tool was also made available (http://cfci.shinyapps.io/ASGA_3DViewer/). The usage of this ASGA tool is shown in Figure 14A–14F and Video 4. In short, upon opening the online tool, the user is welcome to the simple and intuitive GUI to enter the main tool and select the data set of interest. After quick pre-loading of the selected data set, the user can interactively visualize the entire spindle data set, choose from different visualization options, and perform an overlay color-coded analysis.
  **Figure 14.**
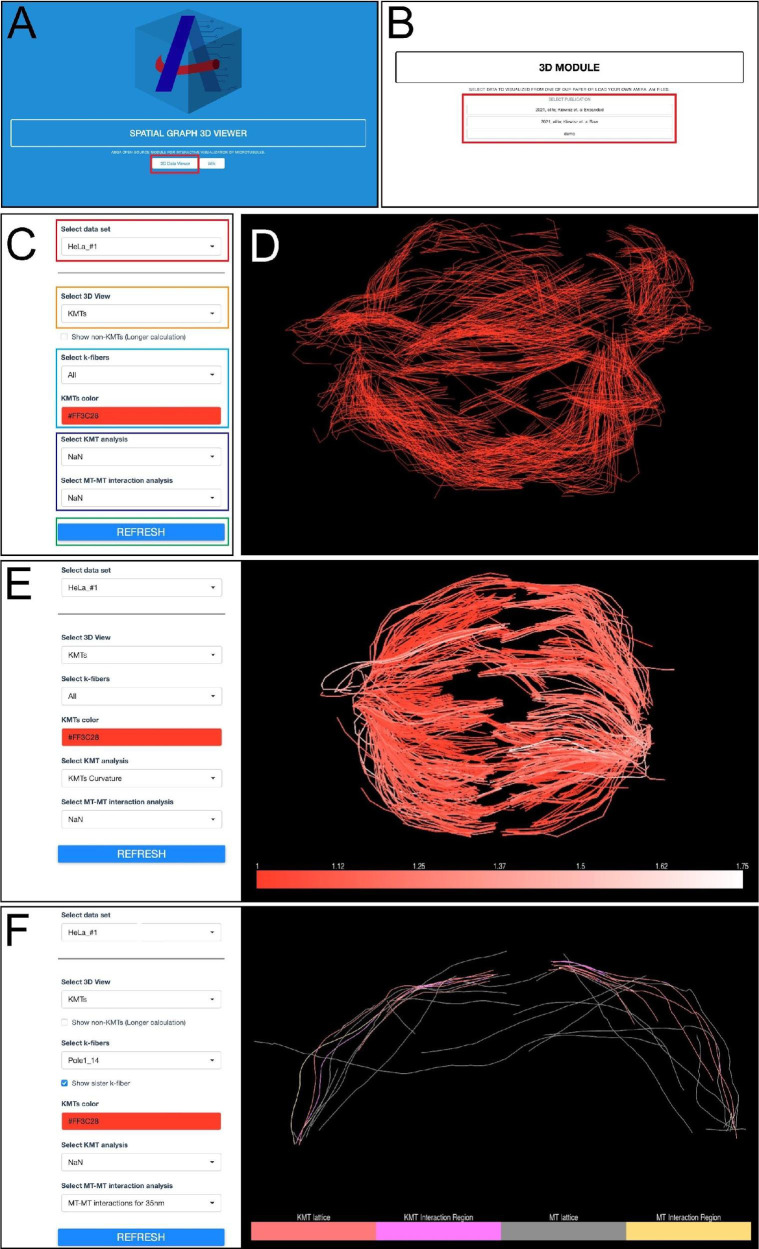
Workflow for microtubule visualization using the ASGA-3DViewer web server. (A) Home screen view of the ASGA-3DViewer. The red box indicates the button initiating the tool. (B) Screenshot of the ASGA-3DViewer indicating the selection of the visualized data set. The red box indicates different data sets from which users can choose. (C) View of ASGA-3DViewer setting panel. The red box shows the selection of the sub-dataset available in the selected collection. The orange box indicates the selection of which type of microtubule class is currently visualized. By default, the ASGA-3DViewer shows only KMTs. However, all microtubules can also be selected. The light blue box indicates the k-fiber and color that is displayed. By default, all fibers are shown in red color. The dark blue box indicates the analyses that can be optionally overlaid on the selected microtubules. The green box indicates a button that is used to apply all selected changes. (D) Example of visualization shown in ASGA-3DViewer showing all kinetochore microtubules. (E) Example of the ASGA-3DViewer showing the settings in the left panel and spindle visualization of all KMTs with overlaid analysis in the right panel. (F) Another example from the ASGA-3DViewer showing selected k-fiber (Dataset – HeLa_#1; K-fiber ID - Pole1_14). This illustrates microtubule–microtubule interaction within a 35 nm distance (Kiewisz et al., 2022; see also Video 4).
  **Video 4.**
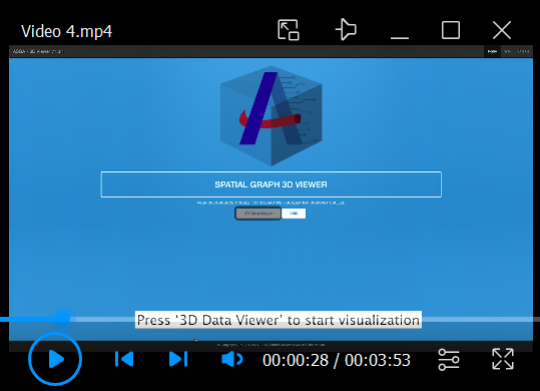
Tutorial video describing the usage of the ASGA-3DViewer software v1.3.1. This video corresponds to Figure 14.

## Validation of protocol

1. Robert Kiewisz, Gunar Fabig, William Conway, Daniel Baum, Daniel Needleman and Thomas Müller-Reichert, (2022). Three-dimensional structure of kinetochore-fibers in human mitotic spindles. *eLife* (11): 1–37. DOI: 10.7554/eLife.75459.
2. William Conway, Robert Kiewisz, Gunar Fabig, Colm P Kelleher, Hai-Yin Wu, Maya Anjur-Dietrich, Thomas Müller-Reichert and Daniel J Needleman, (2022). Self-organization of kinetochore-fibers in human mitotic spindles. *eLife* (11): 1–34. DOI: 10.7554/eLife.75458.
3. Alejandra Laguillo-Diego, Robert Kiewisz, Carlos Martí-Gómez, Daniel Baum, Thomas Müller-Reichert and Isabelle Vernos, (2023). MCRS1 modulates the heterogeneity of microtubule minus-end morphologies in mitotic spindles. *MBoC* (34): 1–14. DOI: 1091/mbc.E22-08-0306-T.

## General notes and troubleshooting

**3D printing:** The designed specimen chambers (Figure 1) can be printed using any commercially available 3D printer supplied with either biodegradable PLA or PLA/PHA plastic. The type of material used for 3D printing should have no effect on the cultivation of the cells. In addition, to avoid any potential risk of contamination with lead, we suggest changing the printing nozzle from the standard brass nozzle to a stainless-steel nozzle.

**Mitotic shake-off:** Make sure to prepare everything in advance that is necessary for the shake-off. The shake-off should be performed as fast as possible to minimize the time during which the cells are exposed to cold temperatures. T75 flasks should always be stored upward (with the lid cup facing upward) to avoid cooling down the cells by the cold bench. Use a long 25 mL pipette to remove or add fresh prewarmed medium to speed up the process. Optionally, you can store a metal plate in the incubator heated up to 37 °C, which can be used to help maintain the temperature of the cell culture.

## Supplementary information

The following supporting information can be downloaded here:

1. File S1. File in.stl format as used for 3D printing of the incubation chambers for a single sapphire disc optimized for performing shake-off experiments. This supplementary information corresponds to Figure 1A.
2. File S2. File in.stl format as used for 3D printing of the incubation chambers for four sapphire discs optimized for performing shake-off experiments. This supplementary information corresponds to Figure 1B.
3. File S3. Protocol for the custom-designed fully automatic generation of tomograms using IMOD.
4. File S4. Detailed explanation of each parameter used for the semi-automatic segmentation of microtubules in Amira. This supplementary information corresponds to Figure 7.
